# Occlusion-Aware Visibility Coverage for Robotic Stop-and-Scan 3D LiDAR Mapping †

**DOI:** 10.3390/s26175430

**Published:** 2026-08-27

**Authors:** Sangmin Kim, Yonghyeon Song, Byeongjun Kim, Tae-Yong Kuc

**Affiliations:** 1 Department of Electrical and Computer Engineering, College of Information and Communication Engineering, Sungkyunkwan University, Suwon 16419, Republic of Korea; kimsang.m@g.skku.edu (S.K.); songyong2535@g.skku.edu (Y.S.); 2 CASELAB Co., Ltd., Anyang 14118, Republic of Korea; byeongjunkim@g.skku.edu; 3 Department of Intelligent Robotics, College of Engineering, Sungkyunkwan University, Suwon 16419, Republic of Korea

**Keywords:** terrestrial laser scanner, LiDAR, stop-and-scan, visibility region, line-of-sight coverage, next-best-view, frontier exploration, 3D mapping, occlusion-aware planning

## Abstract

Automated three-dimensional (3D) mapping with a survey-grade terrestrial laser scanner (TLS) is the canonical instance of robotic stop-and-scan light detection and ranging (LiDAR) mapping: the mobile platform must remain stationary for minutes per scan, so the environment must be covered with as few scans as possible. Where to stop hinges on a coverage model predicting what a scan pose will observe. The conventional isotropic-disk model counts every free cell within sensing range as covered—including cells behind walls—and, therefore, skips scans that genuine observation requires. We replace the disk with a ray-cast visibility region computed on the robot’s live two-dimensional (2D) occupancy map, admitting only cells in direct line of sight; define a line-of-sight (LOS) coverage metric over mapped free space and drive a marginal-gain scan/skip rule embedded in frontier exploration. The planner thus performs 2D scan-station placement for subsequent 3D mapping. In a controlled paired ablation in simulation, the visibility rule raises LOS coverage from about 77% to 84–85% for one to two additional scans, and it also outperforms a disk baseline governed by the identical marginal-gain rule, isolating the coverage model itself as the decisive factor. In a fully autonomous on-hardware comparison in an industrial test room, with each rule driving a mobile platform carrying a Leica BLK360 G1, the visibility rule raised LOS coverage from 77.6% to 92.1%; every visibility run’s scans registered offline at survey grade (4–5 mm bundle error, 84–88% overlap), whereas disk runs yielded at best a single-link network and once a single unregistrable scan. The results indicate that, for the room-scale indoor environments studied, occlusion-aware visibility is a sounder basis than Euclidean proximity for stop-and-scan placement.

## 1. Introduction

Industrial robotics is moving from two-dimensional floor-plan reasoning toward three-dimensional spatial understanding of the work environment. Lift robots, mobile manipulators, and autonomous mobile robots increasingly plan and act in full 3D, and the emerging class of physical-AI systems presumes a metrically faithful 3D model of the space in which they operate. As a result, demand for survey-grade 3D maps of factories, warehouses, and other operational sites is growing. A dense and accurate point cloud is becoming an infrastructural asset: downstream robots localize against it, plan collision-free motion within it, and reason over it. This raises the practical question of how such maps can be acquired automatically rather than through labor-intensive manual scanning. The motivation is threefold: a manual survey occupies a trained operator for hours of tripod placements; a robotized acquisition can run outside operating hours, interrupting neither production processes nor people on the floor and an autonomous procedure makes periodic re-scans repeatable at low marginal cost.

Automated three-dimensional (3D) mapping of indoor and industrial environments is increasingly carried out by mobile robots equipped with survey-grade stationary sensors. We refer to this regime as *robotic stop-and-scan LiDAR mapping*: a mobile platform carries a light detection and ranging (LiDAR) sensor that must remain stationary during each acquisition, and the dense scans are aligned offline rather than registered incrementally in real time. A terrestrial laser scanner (TLS) is the canonical instance of such a sensor; the Leica BLK360 G1 [1] (Leica Geosystems AG, Heerbrugg, Switzerland) used in our experiments acquires a dense point cloud from a single viewpoint with a 3D point accuracy on the order of millimeters and a full-dome scan time of a few minutes. (The scanner also records panoramic RGB imagery that colorizes the point cloud; the planning and evaluation in this paper concern the geometric measurements only.) Two properties shape the mapping problem. First, the sensor must remain completely stationary during acquisition; any base motion introduces distortion and registration error. Second, the dense scans are not registered in real time but aligned offline. We emphasize that these two properties characterize the survey-grade stop-and-scan class, not 3D laser scanning in general: continuous mobile-mapping scanners acquire under motion and register incrementally online through simultaneous localization and mapping (SLAM), trading survey-grade accuracy for acquisition speed. SLAM-based continuous mobile mapping, therefore, does not transfer directly to the stop-and-scan workflow addressed here. The method developed in this paper is formulated for the general stop-and-scan LiDAR regime; the BLK360 G1 enters only as the experimental instrument (Section 5).

A broader class of sensors shares this workflow in that they require stationary acquisition or a dwell time at each viewpoint, including structured-light 3D scanners, thermal panorama units, ground-penetrating radar (GPR), gas and spectral sensors, and non-destructive testing probes [2,3,4]. These instruments differ widely in observation geometry, resolution, and uncertainty; the line-of-sight coverage model developed here applies to the optical, non-penetrating members of this family and explicitly not to penetrating modalities such as GPR (Section 7). Whenever each stop incurs a sensing cost of seconds to minutes, the planner must decide *where* to stop and scan so that the environment is covered with as few stationary acquisitions as possible, while preserving the inter-scan overlap that offline registration requires.

At the heart of this decision is a *coverage model*: a prediction of which part of the environment a scan pose will actually observe. The choice of coverage model determines whether a candidate pose is judged redundant (and skipped) or informative (and scanned). The conventional and computationally convenient choice is an *isotropic disk* model, which treats every free cell within the sensing range as covered—a sensor-footprint approximation widely used to drive online decisions in frontier exploration and coverage practice [5,6,7,8,9]. This assumption is reasonable in open space but fails in the presence of occlusion: it claims cells that lie within range yet are separated from the sensor by a wall or partition, and consequently it judges occluded regions as already covered and skips the scans needed to observe them. In a multi-room environment, this can leave an entire room behind a partition unscanned while the planner believes it is covered.

In this paper, we develop a coverage model and placement policy built on occlusion-aware visibility, and we evaluate whether it provides a sounder basis for stop-and-scan placement than Euclidean proximity. The study is organized around one principal research question:

**RQ.** *In online robotic exploration of an unknown indoor environment with a stop-and-scan LiDAR sensor, does an occlusion-aware visibility coverage model produce better scan placement—higher genuinely observed coverage at a comparable number of stationary scans—than the conventional proximity-based disk model?*

Three subquestions structure the investigation: (RQ1) how should per-pose coverage be defined on the robot’s live 2D occupancy map so that it never claims occluded or unmapped space (Section 3); (RQ2) how much of the placement difference is attributable to the coverage model alone, once exploration stochasticity and the choice of acceptance rule are controlled for (Section 4 and Section 6) and (RQ3) does the effect carry over to a physical platform and to the registered survey product (Section 6.6 and Section 6.7).

Concretely, we replace the disk with a *ray-cast visibility region* that admits only cells in direct line of sight of the scan pose, and we define a *line-of-sight (LOS) coverage* metric as the fraction of mapped free space observed from at least one scan pose; both are computed on the live 2D occupancy grid, so the method performs 2D scan-station planning for subsequent 3D mapping (Section 3.5). The visibility region drives a marginal-gain scan/skip rule that is embedded in a frontier-exploration loop, together with an optional coverage-completion phase that drives residual occluded pockets toward full coverage. The contributions are as follows:

- an occlusion-aware ray-cast visibility coverage model for stop-and-scan sensing, defined on the live 2D occupancy grid, with a conservative LOS coverage metric over mapped free space (Section 3);
- a marginal-gain scan/skip placement policy, with a dual relative/absolute acceptance criterion and an optional coverage-completion phase, embedded in autonomous frontier exploration (Section 4);
- a controlled, confound-free evaluation in a physics-based simulation built from a real scanner-derived environment, isolating the effect of the coverage model from exploration stochasticity and—via a disk baseline run under the identical marginal-gain rule—from the acceptance rule, together with a parameter sensitivity analysis (Section 5 and Section 6);
- a fully autonomous on-hardware disk-versus-visibility comparison on a four-wheeled mobile platform carrying a Leica BLK360 G1 in an industrial test room, with each skip rule driving the platform’s live exploration and scan acquisition, reproducing the placement gain on the physical robot (Section 6.6);
- per-run offline registration of the autonomously acquired BLK360 scans, showing that every visibility run registers at survey grade (4–5 mm bundle error, 84–88% overlap, redundant multi-link networks) while disk runs yield at best a single-link network and at worst a single unregistrable scan (Section 6.7).

This work extends a preliminary conference paper [10], accepted for presentation at ICCAS 2026, and the overlap between the two is as follows. The conference version contributes the stop-and-scan system integration (frontier exploration coupled to a scan sequencer) and an isotropic-disk scan trigger, demonstrated in simulation. New to this article are the ray-cast visibility coverage model and the LOS coverage metric (Section 3); the dual-criterion marginal-gain placement rule and the coverage-completion phase (Section 4); the controlled paired ablation with its three baselines and the parameter sensitivity analysis (Section 6) and the entire hardware study—the fully autonomous on-hardware disk-versus-visibility comparison and the per-run offline registration of the acquired scans (Section 6.6 and Section 6.7). No figure, table, or quantitative result is shared between the two manuscripts.

The remainder of this article is organized as follows. Section 2 reviews related work and states the research gap. Section 3 defines the visibility coverage model and the LOS metric, and Section 4 the placement policy built on it. Section 5 describes the platforms, environments, and parameters. Section 6 reports the simulation study, the on-hardware comparison, and the registration analysis. Section 7 discusses limitations, and Section 8 concludes.

## 2. Related Work

### 2.1. Autonomous Exploration and Next-Best-View Planning

Autonomous exploration of unknown environments has been studied principally under two paradigms [5,11]. Frontier-based methods drive the robot toward the boundary between known and unknown space [6,7], prioritizing frontiers by expected information gain and travel cost. Next-best-view (NBV) methods instead evaluate candidate viewpoints directly by an information-gain or coverage criterion [12,13], selecting the pose that maximizes expected new observation; information-theoretic formulations make this gain explicit by quantifying the expected reduction in map uncertainty [14], and the idea extends naturally to the coordination of multiple robots [15]. Both paradigms have been refined extensively, for example, through improved frontier extraction and cost shaping [16,17], incremental frontier structures with hierarchical planning [18], and sampling-based online informative path planning [19]. Most of these methods, however, assume a sensor that acquires continuously while the platform moves and that registers incrementally online, so the dominant cost is travel and the act of “observing” is effectively free along the path. Under that assumption, the question of whether to pay a high fixed cost to stop and observe at a particular pose does not arise.

### 2.2. Coverage Models and Visibility

The notion that a viewpoint observes only what is in its line of sight is classical in computational geometry, where the visibility polygon defines the region directly visible from a point, and in the art-gallery problem, which asks for a minimal set of guards that jointly see an entire domain [20]. The art-gallery problem and its many variants have been studied extensively in computational geometry [21,22]. Sensor-coverage and view-planning formulations build on this idea to place viewpoints that maximize observed surface, and they have been surveyed for automated 3D reconstruction and inspection [23], for sensor planning in computer vision [24], and, more recently, for view planning in robot active vision at large [25]. Coverage path planning, which instead seeks a path that sweeps a sensor footprint over an entire area, has likewise been surveyed extensively [8,9], including for multi-robot model reconstruction and mapping [26]. In robotic exploration practice, however, the per-pose coverage used to drive online decisions is frequently approximated by a range disk or a sensor cone without explicit occlusion reasoning, because such approximations are cheap to evaluate at every candidate. This approximation is benign when observation is continuous and cheap, but it becomes consequential precisely in the stop-and-scan regime, where a single skipped occluded region corresponds to a missing multi-minute scan and an unobserved part of the map.

### 2.3. Stationary and Stop-and-Scan Mapping

Survey-grade terrestrial scanning, as practiced with the tripod-class instruments considered here, departs from the continuous-acquisition assumption: each measurement requires a stationary dwell of minutes and registration is performed offline [2]. Scanners that relax these constraints exist, but at accuracies below the survey grade targeted here. Prior automated TLS and stop-and-go mapping efforts focus largely on the system integration of driving a scanner between viewpoints and on offline registration of the collected scans [27], while scan-planning studies in construction optimize viewpoint placement to maximize the visible area of target structures [28]. Some of these planning methods are explicitly occlusion- or visibility-aware: Mozaffar and Varshosaz [29] optimize scanner placement to reduce occlusions, and Prieto et al. [30] drive a next-best-scan policy with a line-of-sight visibility analysis. Recent formulations cast indoor scan planning as combinatorial optimization over a known floor plan: Dehbi et al. [31] compute minimal TLS viewpoint sets under visibility and registrability (network-connectivity) constraints, and Knechtel et al. [32] jointly optimize stop-and-go standpoints and the route between them with enforced network redundancy. Crucially, however, these methods operate *offline on a prior model* of the environment (a building information model (BIM), floor plan, or an initial coarse scan), over which candidate viewpoints and their visibility are evaluated in advance. The decision of which candidate poses are worth a stationary scan during *online* exploration of an *unknown* space is still typically handled by fixed spacing or by a proximity (disk) test. The present work targets exactly that decision: it makes the online coverage model occlusion-aware via ray-cast visibility on the live map, so that the scan/skip choice reflects what the scanner can genuinely observe rather than what merely lies within range. This complements frontier and NBV exploration rather than replacing it; the visibility model supplies the coverage estimate, while a frontier explorer supplies navigation goals.

### 2.4. Positioning of This Work

The two bodies of work above leave a specific gap. NBV and frontier exploration operate *online* in unknown environments but assume continuous, low-cost acquisition, so they do not reason about whether stopping to observe is worth a high fixed cost. Automated TLS and scan-planning methods do reason about expensive stationary acquisition, but they are typically *offline* and assume a known environment model (e.g., a BIM or floor plan) over which viewpoints are optimized in advance. The regime addressed here is the intersection of the two: *online* exploration of an *unknown* environment in which each acquisition carries a *large stationary cost*, where the scan/skip decision must be made on the live map. In that regime the coverage model is the decisive component, and we argue that it must be occlusion-aware rather than proximity-based: an isotropic-disk or fixed-spacing rule, a commonly used online treatment in stop-and-go practice, over-claims coverage behind walls and suppresses necessary scans. Replacing that rule with a ray-cast visibility model is the contribution of this paper.

## 3. Visibility-Based Coverage Model

This section defines the coverage model at the core of the method. We formalize the workspace on an occupancy grid (Section 3.1), contrast the isotropic disk model (Section 3.2) with the proposed ray-cast visibility region (Section 3.3), and define the line-of-sight coverage metric (Section 3.4).

### 3.1. Workspace and Notation

Let the environment be represented by a two-dimensional occupancy grid [33,34] M produced online by SLAM, following the nav_msgs/OccupancyGrid convention. The grid is an H×W array of square cells of side ρ, the grid resolution (ρ=0.05 m in all experiments); each cell *c* is identified with its center point in the map frame, so that $∥c-c^{\prime }∥$ denotes the Euclidean distance between cell centers. Each cell carries an integer value o(c), where o(c)=−1 denotes unmapped (unknown) space and o(c)∈[0,100] is a scaled occupancy probability. Cells are classified as

(1) $$\mathrm{state}\left(c\right)=\left\{\begin{array}{cc} \mathrm{free}, & 0\leq o\left(c\right)\leq \theta_{\mathrm{free}},\\ \mathrm{occupied}, & o\left(c\right)\geq \theta_{\mathrm{occ}},\\ \mathrm{unknown}, & o\left(c\right)=-1\;\mathrm{or}\;\theta_{\mathrm{free}}<o\left(c\right)<\theta_{\mathrm{occ}}, \end{array}\right.$$

with thresholds ordered as $0\leq \theta_{\mathrm{free}}<\theta_{\mathrm{occ}}\leq 100$; we use $\theta_{\mathrm{free}}=25$ and $\theta_{\mathrm{occ}}=65$, the default free/occupied probability thresholds (0.25/0.65) of the Nav2 map server used in our stack (nav2_map_server; https://github.com/ros-navigation/navigation2, accessed on 23 August 2026). The classification is insensitive to the exact placement of these values because SLAM-estimated occupancies are strongly bimodal—almost all mapped cells settle near 0 or near 100—so any thresholds separating the two modes yield the same classification. The two thresholds are deliberately separated: a cell is treated as free only when its occupancy is confidently low ($\leq \theta_{\mathrm{free}}$) and as an obstacle only when confidently high ($\geq \theta_{\mathrm{occ}}$); the intermediate band is treated as unknown so that ambiguous cells are never counted as covered nor allowed to pass a sight line. We write F={c∈M:state(c)=free} for the set of mapped free cells, the region over which coverage is evaluated.

A *scan pose* is a stationary viewpoint $s_{i}\in F$ at which the platform halts and the stop-and-scan sensor acquires data. Each sensor is characterized by a range bound *R*, the maximum distance out to which its visibility region is evaluated. For a set of *n* scan poses $S=\{s_{1},\ldots ,s_{n}\}$, the coverage model quantifies which portion of F is genuinely observed. Throughout the paper, $s_{i}$ denotes an accepted scan pose and *p* a candidate pose under evaluation (Section 4), while *c* is reserved for grid cells.

### 3.2. Isotropic Disk Coverage (Baseline)

**Figure 1:**
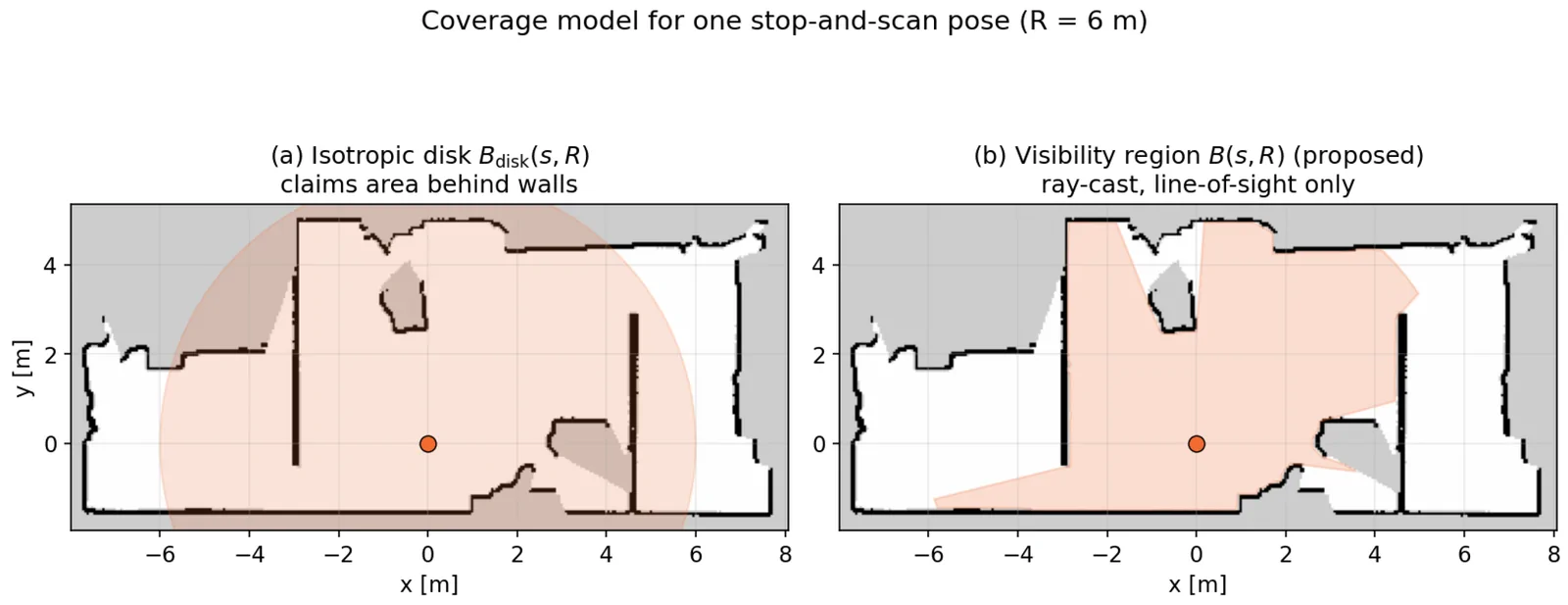
Coverage models for a single scan pose (R=6 m). The orange dot marks the scan pose *s*. (**a**) The isotropic disk $B_{\mathrm{disk}}\left(s,R\right)$ counts every free cell within range as covered, bleeding through walls. (**b**) The proposed ray-cast visibility region B(s,R) claims only genuine line-of-sight area.

A widely adopted and computationally convenient assumption is that a scan pose covers every free cell within its range, giving the *isotropic disk* model

(2) $$B_{\mathrm{disk}}\left(s_{i},R\right)=\left\{c\in F:∥c-s_{i}∥\leq R\right\},$$

where ∥·∥ is Euclidean distance. The disk model depends only on the range bound and is trivial to evaluate, and it is adequate in open, obstacle-free space. Its defining limitation is that it ignores occlusion: because membership depends solely on proximity, $B_{\mathrm{disk}}$ claims cells within range *R* that are separated from $s_{i}$ by a wall or partition, i.e., cells to which the sensor has no line of sight. In structured indoor and industrial environments this asserts coverage of space *behind* obstacles that the sensor cannot observe (Figure 1a). At the placement stage, a rule that trusts $B_{\mathrm{disk}}$ treats such a region as satisfied and skips the scan that genuine observation would require (Section 4).

### 3.3. Ray-Cast Visibility Region (Proposed)

To account for occlusion, we replace the disk with a *ray-cast visibility region*, a discrete visibility polygon that admits only cells in direct line of sight of the scan pose. From $s_{i}$, K=720 rays are cast over a full $360^{\circ }$ field at uniform angular resolution $\Delta \theta =360^{\circ }/K=0.5{}^{\circ}$. Each ray is marched outward from $s_{i}$ in steps of half a cell and is truncated at the first sample that is either occupied or unknown; that terminating cell and everything beyond it on the ray are excluded. Formally, for a free cell *c* on the ray at azimuth $\theta_{k}$, let $\bar{s_{i}c}$ denote the cells strictly between $s_{i}$ and *c* along the ray. Then

(3) $$B\left(s_{i},R\right)=\left\{c\in F:∥c-s_{i}∥\leq R\;\wedge \;\mathrm{state}\left(c^{\prime }\right)=\mathrm{free}\;\;\forall \;c^{\prime }\in \bar{s_{i}c}\right\}.$$

A free cell is claimed as covered only if every intervening cell along the sight line to it is also mapped to free space. The ray stops at the first occupied or unknown cell: occupied cells ($o\geq \theta_{\mathrm{occ}}$) terminate the ray because the obstacle blocks the beam, and unknown cells terminate it because the model refuses to assert visibility through unmapped space. This second condition is deliberately conservative; the model never “sees through” regions the map cannot vouch for, so $B(s_{i},R)$ reports only observation that is geometrically defensible given the current map. The same stopping rule is applied uniformly across the model, the implementation, and every experiment in this paper. Note that the set definition above characterizes the ideal geometric visibility region and does not depend on the ray discretization; the *K* rays and half-cell marching are the implementation that approximates it. At the range bound, the inter-ray arc spacing is RΔθ≈5.2 cm for R=6 m, on the order of a single grid cell (ρ=5 cm), so the discrete approximation recovers the ideal region up to single-cell effects at region boundaries.

By construction $B\left(s_{i},R\right)\subseteq B_{\mathrm{disk}}\left(s_{i},R\right)$ for any pose and range: ray-casting can only remove cells the disk would have claimed, never add new ones, and the two coincide only for an obstacle-free disk around $s_{i}$. Figure 1 contrasts the two: where the disk (Figure 1a) bleeds through partition and perimeter walls, the visibility region (Figure 1b) is clipped to the genuinely observable area. Each visibility polygon is computed in pure array operations in roughly 2 ms, inexpensive enough to evaluate at every scan candidate online.

### 3.4. Line-of-Sight Coverage Metric

Coverage by a set of scan poses is the union of their visibility regions. For $S=\{s_{1},\ldots ,s_{n}\}$ the covered area is

(4) $$C\left(S\right)=\overset{n}{\underset{i=1}{⋃}}B\left(s_{i},R\right),$$

and the *line-of-sight (LOS) coverage* is the fraction of mapped free space observed from at least one scan pose,

(5) $$\mathrm{LOS}\left(S\right)=\frac{\left|C(S)\cap F\right|}{\left|F\right|}\in \left[0,1\right],$$

where |·| counts cells. LOS coverage measures the proportion of mapped free space for which the sensor set guarantees direct line of sight; the complement 1−LOS(S) corresponds to mapped free cells that remain occluded from every scan pose. Because the metric is defined over F and visibility never propagates through occupied or unknown cells, LOS coverage is a conservative measure of genuine observation rather than the optimistic proximity count produced by the disk model. It is the single figure of merit reported throughout the experiments.

### 3.5. Scope: 2D Station Planning for 3D Mapping

The visibility model and the LOS metric are deliberately two-dimensional: they reason on the live occupancy grid, a horizontal slice of the environment at the mapping sensor’s height. LOS coverage, therefore, certifies sight lines to floor-space cells, not to the full 3D surface set the scanner observes. Vertical occlusions—shelves, overhanging structures, elevated machinery, or surfaces stacked at different heights above the same floor cell—are not represented, and high 2D LOS coverage does not by itself guarantee complete 3D surface coverage. This scope is a design choice matched to the online setting: the occupancy grid is what SLAM makes available in real time on the platform; the 2D visibility polygon is cheap enough to evaluate at every candidate and in the room-scale indoor environments targeted here, a pose with unobstructed 2D line of sight to a floor region generally also observes much of the structure above it, because the scanner acquires a full dome. The contribution of this paper should accordingly be read as occlusion-aware *2D scan-station planning for subsequent 3D mapping*; extending the model to a genuinely 3D visibility representation (voxel-, octree-, or surface-based) is future work (Section 7).

## 4. Visibility-Based Stop-and-Scan Placement

Section 3 defined what a scan pose observes; this section defines where the robot decides to stop and scan. Because each stationary scan carries a large fixed cost (minutes per acquisition for the survey-grade scanner used in our experiments; Section 5), the placement policy must take as few scans as possible while maintaining high LOS coverage of the mapped free space.

### 4.1. Marginal Visibility Gain

As the robot travels, a scan candidate is raised every *d* meters of net straight-line displacement in the map frame from the previous candidate pose (the evaluation reference, initialized to the start pose and advanced at every raised candidate whether or not it is accepted; net displacement rather than path length, so dwelling or circling in place raises no candidate). Let $S=\{s_{1},\ldots ,s_{m}\}$ be the scans accepted so far and $C\left(S\right)={⋃}_{i}B\left(s_{i},R\right)$ their union coverage. The visibility regions of previously accepted scans are not frozen: at every decision, C(S) is recomputed on the current occupancy map, so earlier regions expand or contract as SLAM refines the map (the final reported coverage is evaluated once more on a single reference map; Section 5). For a candidate pose *p*, the decision is based on how much *new* line-of-sight area its visibility region contributes relative to what it sees in total. We define the *normalized marginal gain*

(6) $$g\left(p|S\right)=\frac{\left|B(p,R)\setminus C(S)\right|}{\left|B(p,R)\right|},\;g\in \left[0,1\right],$$

the fraction of the candidate’s own visibility region not already covered, and the absolute new area

(7) $$a\left(p|S\right)=\left|B\left(p,R\right)\setminus C\left(S\right)\right|\cdot \rho^{2},$$

where cells are squares of side ρ (the grid resolution, ρ=0.05 m), so each cell contributes area $\rho^{2}$ and *a* is in m^2^. Both quantities are defined only for a nonempty visibility region B(p,R), and are evaluated directly on the visibility regions of Section 3, so occlusion is respected: area behind a wall is never part of B(p,R) and never inflates either the coverage or the gain. A candidate whose visibility region is degenerate (smaller than 0.5 m^2^, which occurs when the pose projects into an obstacle or an unmapped pocket where almost every ray is immediately blocked) is rejected outright before the gain is evaluated, so the denominator of the normalized gain never vanishes.

### 4.2. Dual-Criterion Accept/Skip Rule

A naive policy would accept a candidate whenever its relative gain exceeds a threshold, g≥τ. This single relative test is fragile at the spatial scale of a room-scale stop-and-scan sensor: because *R* is comparable to the size of a room, the region B(p,R) is large, so a small but genuinely occluded pocket contributes only a tiny *fraction* of that large region and fails the relative test, even though it is exactly the kind of unscanned area the method exists to capture. We, therefore, accept a candidate when *either* a relative *or* an absolute criterion is met:

(8) $$\mathrm{scan}\left(p\right)\Leftrightarrow \left(g(p|S)\geq \tau \right)\;\vee \;\left(a\left(p|S\right)\geq A_{\min}\right),$$

and skip it only when both fail. The relative term τ captures candidates that open a large new area such as a previously unseen room; the absolute term $A_{\min}$ rescues small but real occlusion pockets whose new area is a negligible fraction of the scanner’s footprint. Throughout the experiments we use τ=0.30 and $A_{\min}=5$ m^2^ with range bound R=6 m. Section 6 shows that coverage and scan count are nearly flat in τ over [0.10,0.50], whereas $A_{\min}$ is the operative knob trading coverage against scan count, with its knee at 5 m^2^.

Two disk-based baselines are evaluated against this rule. The first is the *proximity-spacing rule* of stop-and-go practice: skip any candidate within *R* (straight line) of an existing scan. We note explicitly that this rule is *not* mathematically equivalent to the marginal-gain rule applied to $B_{\mathrm{disk}}$—a candidate closer than *R* to an existing scan can still contribute a crescent of new disk area—so a comparison against it changes both the coverage model and the acceptance rule. To isolate the coverage model itself, the ablation of Section 6, therefore, additionally evaluates a *disk marginal-gain* baseline that applies the identical dual criterion above with $B_{\mathrm{disk}}$ substituted for *B* (and disk-based bookkeeping of the covered set); within that pair, the only difference is the coverage model.

### 4.3. Coverage-Completion Phase

Frontier-driven exploration terminates when the known map stops growing (Section 4.4), but convergence of the *map* does not by itself guarantee that every mapped free cell has been brought into line of sight: small occluded pockets can remain. The method, therefore, includes an optional coverage-completion phase that runs after exploration converges. The uncovered free cells F∖C(S) are clustered into connected pockets; for each pocket of area at least $A_{\mathrm{pocket}}$, the robot navigates into the pocket and takes a stationary scan, repeating until no qualifying pocket remains. The phase is bounded by per-pocket navigation timeouts, an overall scan budget, and a list of pockets found unreachable, so it always terminates. In practice the method does not claim full 100% LOS coverage: sub-$A_{\mathrm{pocket}}$ fragments at wall and obstacle edges are reported honestly as residual rather than rounded up.

### 4.4. Integration with Exploration

The placement policy is embedded in the autonomous exploration loop without owning the navigation goals. A frontier explorer drives the robot toward unmapped space on the live occupancy map; a sequencer monitors displacement, applies the accept/skip rule at each raised candidate, and triggers a stationary scan when a candidate is accepted, resuming exploration afterward. Completion is declared by a separate monitor when the count of known cells fails to increase by a minimum margin over a fixed window while the robot is actively exploring, which requires no ground truth. Once exploration is complete, the optional coverage-completion phase runs, after which the run is finalized.

Algorithm 1 summarizes the complete placement policy, combining the marginal-gain evaluation, the dual-criterion accept/skip rule, and the coverage-completion phase within the exploration loop.
**Algorithm 1:** Visibility-based stop-and-scan placement
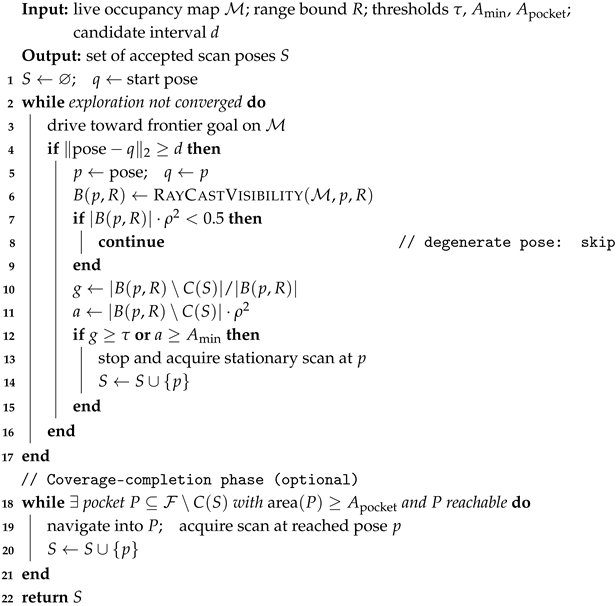


## 5. Experimental Setup

### 5.1. Platform and Software Stack

**Figure 2:**
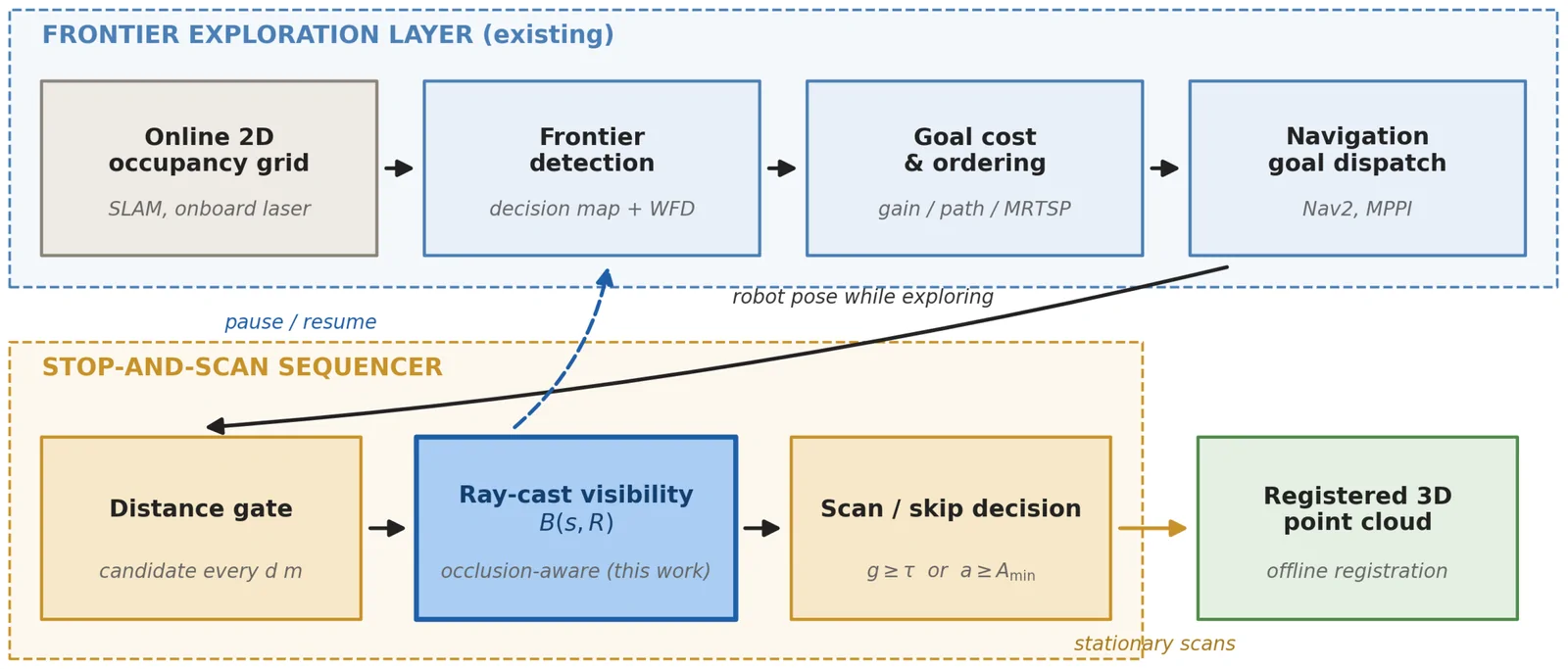
System architecture. The exploration layer (**top**) proposes navigation goals on the live occupancy grid; the stop-and-scan sequencer (**bottom**) applies the visibility-based scan/skip rule (highlighted; this work) and triggers stationary scans that are registered offline.

**Figure 3:**
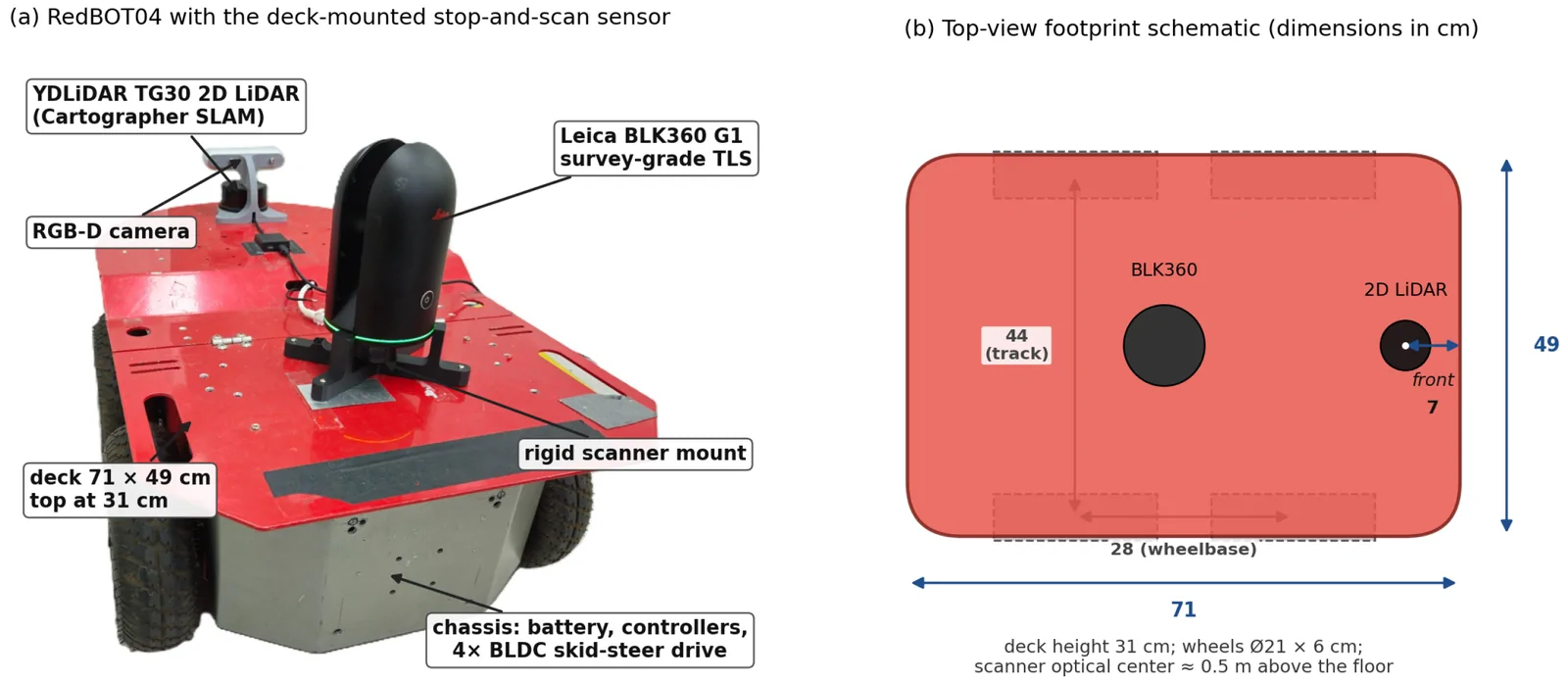
RedBOT04 mobile scan platform. (**a**) Annotated view with the deck-mounted Leica BLK360 G1; (**b**) top-view footprint schematic with the principal dimensions (cm).

The method is implemented as a set of decoupled ROS 2 (https://www.ros.org, accessed on 23 August 2026) nodes. A mobile base builds a 2D occupancy map online with Cartographer SLAM [35] (https://github.com/cartographer-project, accessed on 23 August 2026); a frontier explorer [36] selects goals and drives the robot through the Nav2 navigation stack [37] (https://nav2.org, accessed on 23 August 2026) using a sampling-based model-predictive path integral (MPPI) controller [38] and a stop-and-scan sequencer applies the placement policy of Section 4, triggering a stationary scan whenever a candidate is accepted and gating successive scans so that the long scan-and-download cycle of the scanner never overlaps with the next acquisition. Figure 2 shows how these components connect: the frontier exploration layer proposes and orders navigation goals, and the sequencer arbitrates the stationary scans, with the ray-cast visibility model of Section 3 as the component that decides whether each candidate is scanned or skipped. All navigation is subject to the platform’s physical constraints: Nav2 plans on an inflated costmap, so every path and every scan candidate keeps the robot footprint (Figure 3b) clear of obstacles by the Nav2 costmap inflation margin (inflation radius 0.70 m with a 0.22 m robot radius in all reported runs).

The target stationary sensor is a Leica BLK360 G1 terrestrial laser scanner. The software stack includes a driver that connects to a physical BLK360 G1 over WiFi and executes its full-dome scan workflow, and we verified that the device can be triggered and read back through this path. The controlled coverage evaluation (Section 6) is conducted *in simulation*: a TurtleBot3 Waffle is the mobile base in Gazebo Sim (Harmonic, gz-sim 8.11.0) [39], and the physical scanner is replaced by a mock scanner node that reproduces the BLK360 capture-and-download timing and its failure-and-retry behavior. Simulation is used deliberately for the primary quantitative comparison because it permits a confound-free, paired ablation in which the disk and visibility rules are replayed on *identical* maps and candidate paths (Section 6), which a physical run cannot reproduce exactly.

To complement the simulation and validate the workflow on real hardware, we also built a physical scan platform, RedBOT04, and used it for a fully autonomous on-hardware disk-versus-visibility comparison in an industrial test room, in which each skip rule drives live exploration and scan placement and the deck-mounted BLK360 acquires a stationary scan at every accepted pose (Section 6.6); every run’s scans were then registered offline as independent projects (Section 6.7). RedBOT04 is a four-wheeled skid-steer mobile base (footprint 71×49 cm) driven by four BLDC gearmotors through an MD200 driver under RS-485 (MDROBOT Co., Ltd., Goyang, Republic of Korea), with an Advantech i7 main controller (Advantech Co., Ltd., Taipei, Taiwan) and an STM32 Nucleo-F746ZG sub-controller (STMicroelectronics, Geneva, Switzerland); a YDLiDAR TG30 (Shenzhen EAI Technology Co., Ltd., Shenzhen, China) provides the 2D scan for Cartographer SLAM and Nav2 navigation, and a Leica BLK360 G1 is mounted on the deck as the survey-grade stationary sensor (Figure 3). The deck places the scanner’s optical center roughly 0.5 m above the floor—lower than a typical 1.5–1.8 m tripod setup—a vantage whose consequences are discussed in Section 7.

### 5.2. Environments and Metric

**Figure 4:**
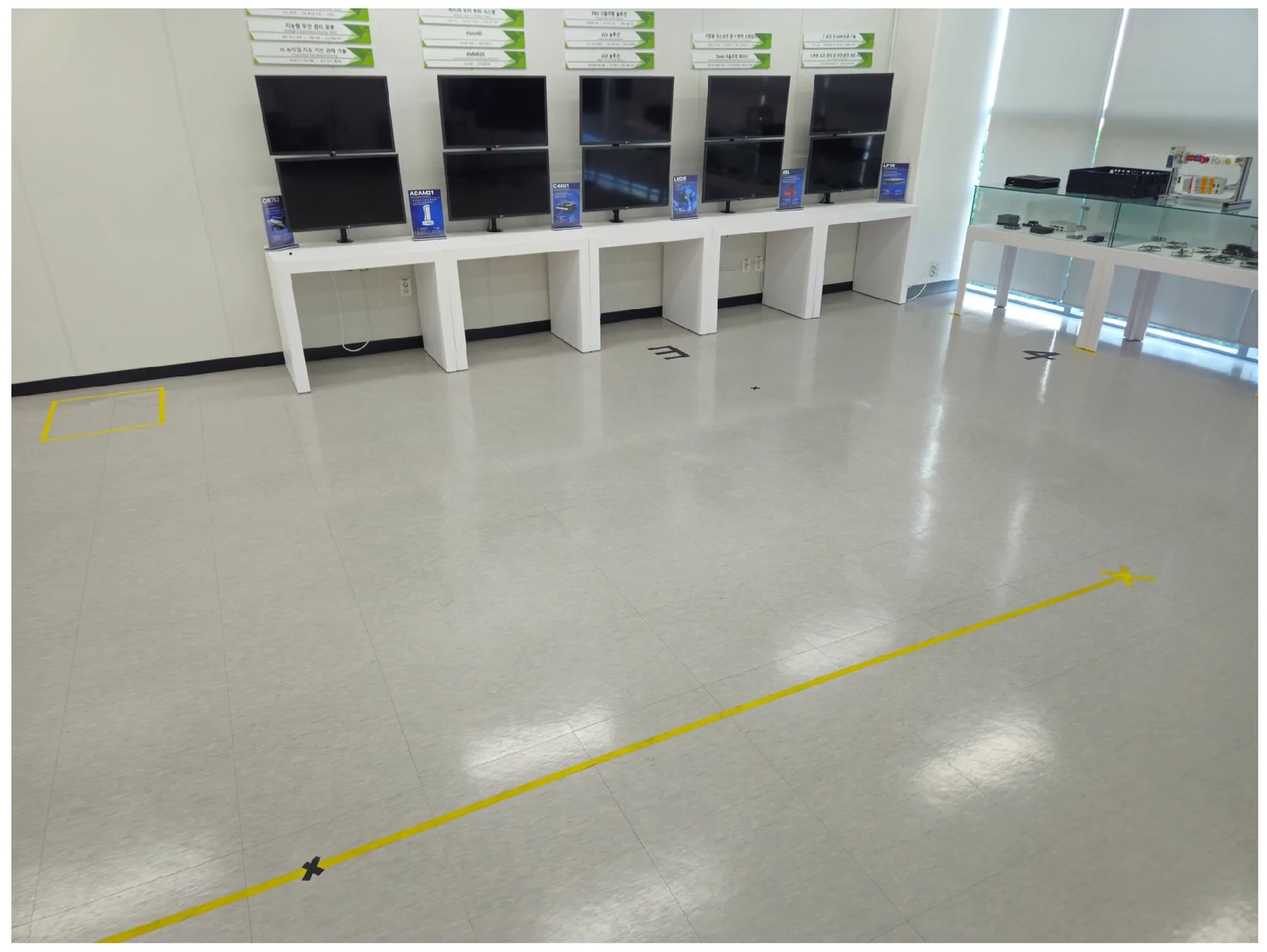
Physical industrial test room used for the real-hardware experiments (Section 6.6 and Section 6.7).

Two indoor worlds are used. The single-room world is generated from a real BLK360 .e57 scan of a laboratory space, converted to a 2D occupancy grid and then to a Gazebo world, so the simulated geometry reflects a real environment. The multi-room world extends this with two interior partitions and offset doorways, producing three rooms with mutual occlusion; it is the setting in which the difference between Euclidean-disk and line-of-sight coverage is most pronounced. For the real-hardware experiments (Section 6.6 and Section 6.7), a physical industrial test room is used. It is an open exhibition and staging space with a flat, reflective floor; a display wall of monitors and product panels runs along one side, and freestanding equipment cases and furniture create genuine self-occlusion. Floor markings indicate the survey reference layout. The room is representative of the factory and demonstration environments the platform targets, and it is shown in Figure 4. Its mapped free space spans approximately 16×8.5 m, about 105 m^2^ of free floor area on the reference map. The figure of merit is LOS coverage (Section 3.4). Because each autonomous run maps a slightly different extent of free space, for any cross-run comparison we re-evaluate every run on a single common reference map (the most complete map among the runs being compared) so that all runs share the same free-space denominator. Scan count is reported alongside coverage as the cost term. Unless noted, experiments use R=6 m, τ=0.30, $A_{\min}=5$ m^2^, a candidate every d=3 m of net displacement, and K=720 rays. Table 1 lists the full parameter set.

## 6. Results

We evaluate the visibility-based policy on two axes, LOS coverage and stationary scan count, organizing the evaluation so that the most rigorous evidence carries the argument. Four placement policies are compared throughout: (i) a *uniform no-skip* reference that scans at every candidate—the fixed-interval policy of stop-and-go practice and an upper reference on the coverage attainable along a path; (ii) the *disk spacing* baseline, the proximity rule of stop-and-go practice; (iii) the *disk marginal-gain* baseline, which applies the identical accept/skip rule as our policy to the disk coverage model (Section 4) and (iv) the proposed *ray-cast visibility* policy. The disk baselines represent a commonly used proximity-based online coverage treatment in stop-and-go practice (Section 2); published TLS scan-planning methods are not applicable as online baselines because they optimize viewpoints offline over a prior environment model [28,31,32], whereas the regime studied here requires every decision to be made on the live map of an unknown environment. The simulation study comes first; Section 6.6 then reports a fully autonomous on-hardware disk-versus-visibility comparison in a physical industrial test room, with scans acquired live, and Section 6.7 closes the loop by registering every run’s scans offline, run by run.

### 6.1. Coverage Model and Single-Room Placement

**Figure 5:**
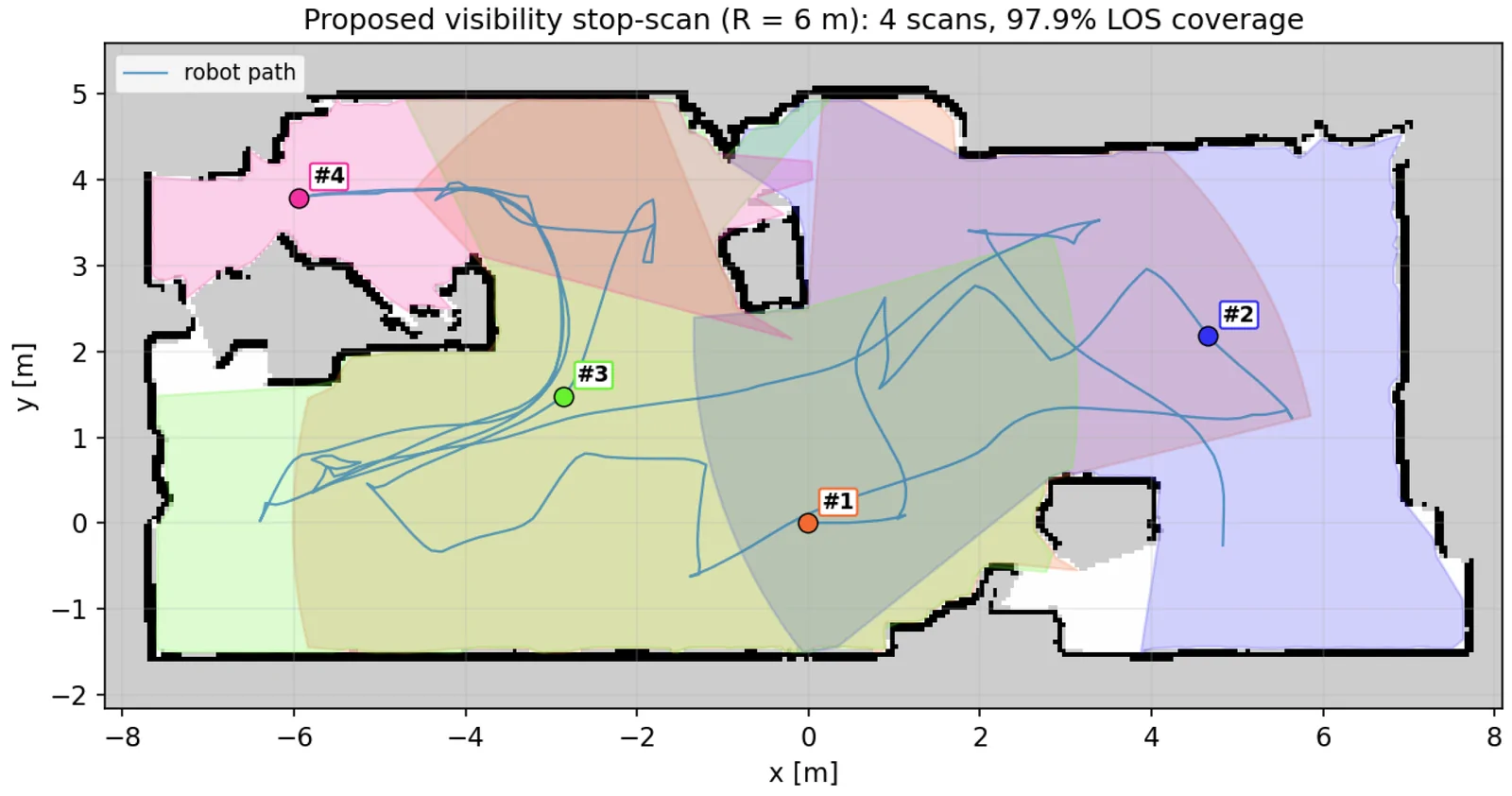
Proposed visibility stop-and-scan on the single-room world (R=6 m): 4 scans, 97.9% LOS coverage. Each accepted scan pose (marker) and its visibility polygon share one color, indexed by scan order (#1…#4); the grayscale background is the occupancy map (light gray free, black occupied, darker gray unknown) and the blue line is the robot path. The same color convention is used in all placement figures.

**Figure 6:**
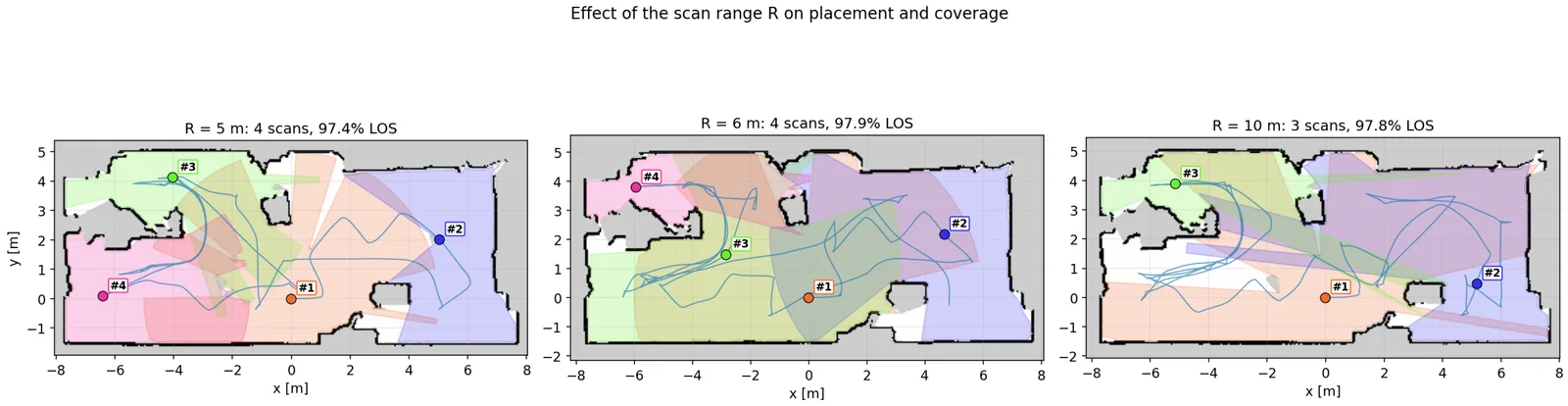
Effect of the range bound *R* on placement and coverage for R=5/6/10 m. Larger *R* needs fewer scans but spreads them; R=6 m balances coverage (97.9%) against inter-scan overlap for registration. Markers #1–#4 and colors index the scan order, as in Figure 5.

Figure 1 contrasts the two coverage models for a single scan pose: the disk claims area on the far side of partition and perimeter walls, whereas the ray-cast visibility region is clipped to genuine line of sight. On the full single-room world, the visibility stop-and-scan policy places four scans and reaches 97.9% LOS coverage (Figure 5). Varying the range bound R∈{5,6,10} m yields 97–98% LOS in all cases (Figure 6); larger *R* needs fewer scans but spreads them apart, reducing inter-scan overlap. We adopt R=6 m for three reasons: it balances coverage against the scan-to-scan overlap that downstream point-cloud registration benefits from; it keeps every cell claimed as covered well inside the scanner’s highest-accuracy band, since the BLK360 G1 specifies a 3D point accuracy of 6 mm at 10 m (8 mm at 20 m) [1] and it matches the local room/bay scale, and hence the occlusion scale, of the indoor environments targeted here.

### 6.2. Controlled Skip-Rule Ablation (Primary Result)

**Figure 7:**
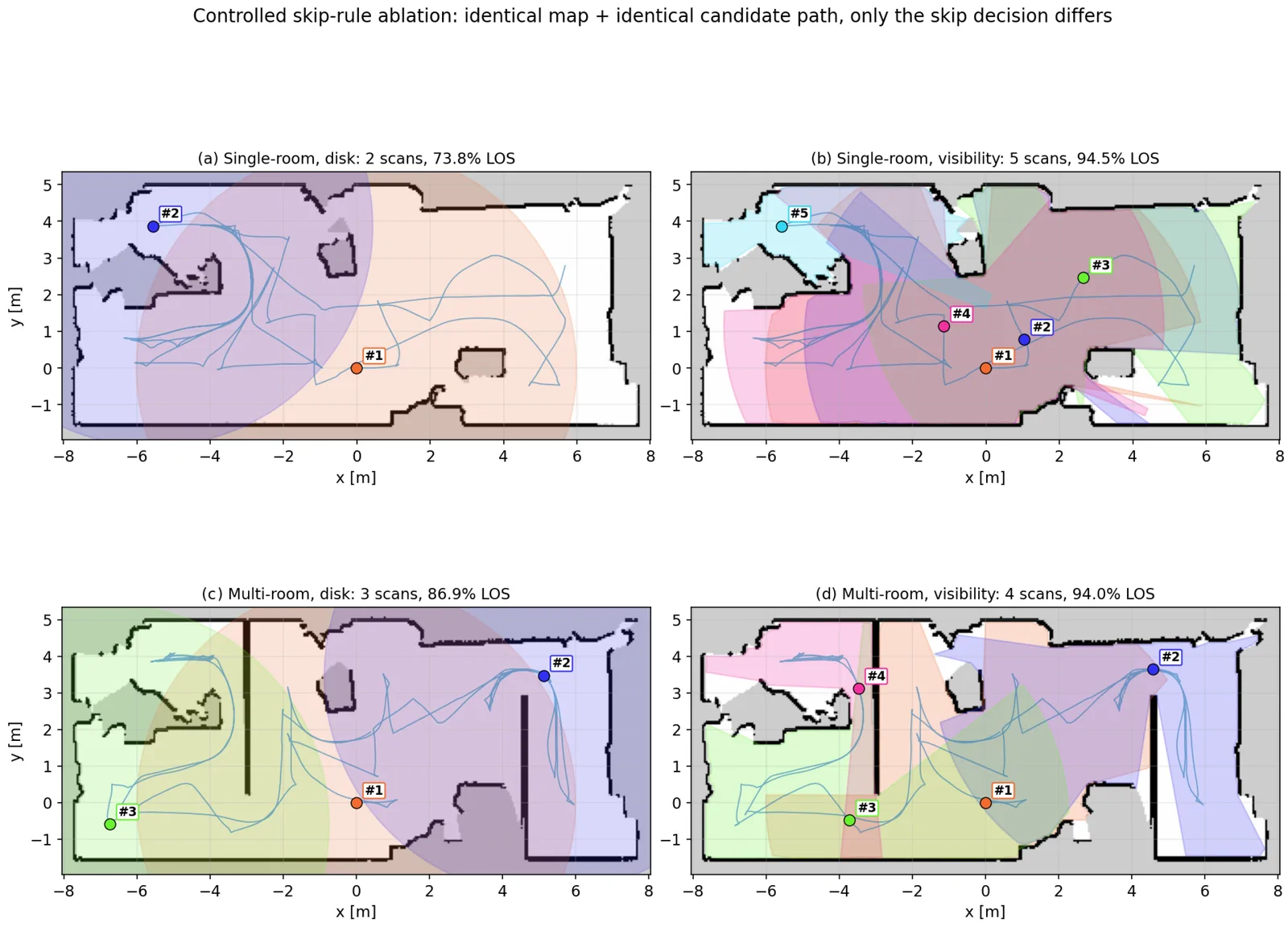
Controlled skip-rule ablation on an identical map and candidate path; only the skip decision differs. (**a**,**b**) Single-room and (**c**,**d**) multi-room, under the disk spacing rule (**a**,**c**) and the visibility rule (**b**,**d**). Markers #1–#5 and colors index the scan order, as in Figure 5.

The decisive comparison isolates the only variable of interest, the accept/skip decision, from the stochasticity of autonomous exploration. On a fixed environment map and a fixed candidate sequence (a logged trajectory sampled every 2 m of path length), we replay both skip rules, so the disk and visibility policies see identical maps and identical candidate poses and differ only in which candidates they accept (Figure 7). For the controlled replay only, each logged trajectory is resampled at fixed 2 m path-length intervals to construct the common candidate sequence: path-length resampling of a logged trajectory yields a deterministic sequence shared by all policies, and the interval is chosen denser than the live d=3 m net-displacement trigger so that no policy is starved of decision opportunities relative to a live run. This deterministic, paired design removes the confound that, in live runs, which rooms get mapped is dominated by navigation through narrow doorways rather than by the skip rule.

**Figure 8:**
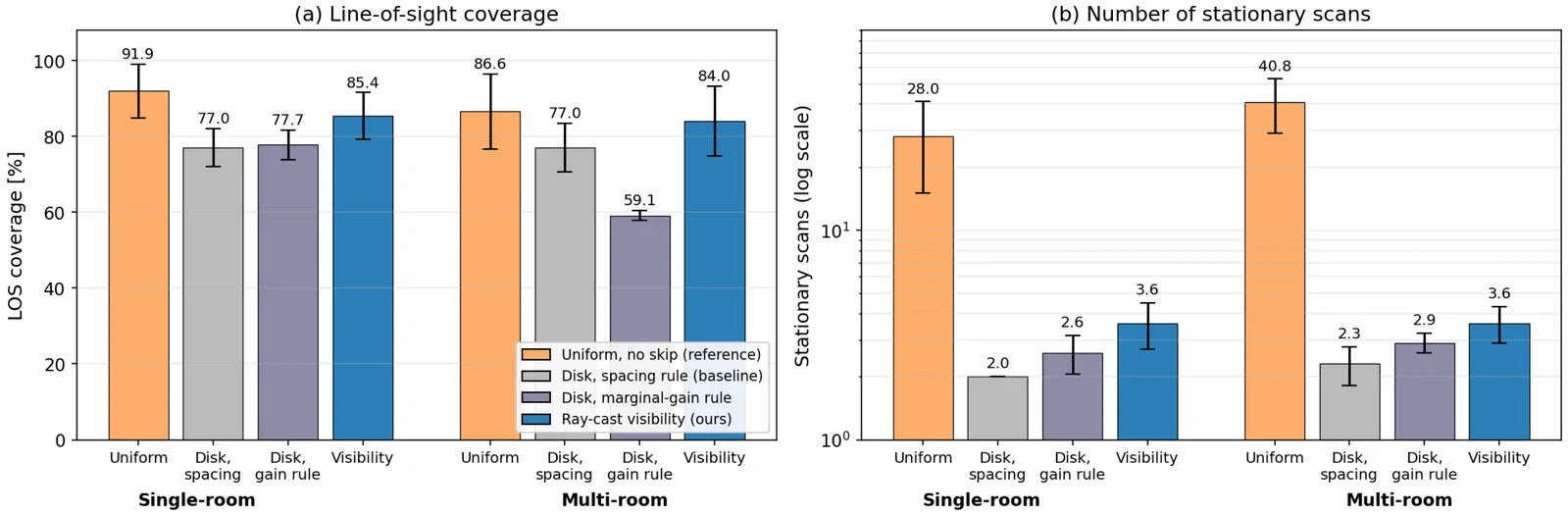
Four-way skip-rule comparison on identical paths (N=5 single-room and N=10 multi-room trajectories, paired per path). (**a**) LOS coverage; (**b**) stationary scan count, log scale (mean ± s.d.).

Across N=5 single-room and N=10 multi-room trajectories (five per coverage model), the visibility rule raises LOS coverage from 77.0±4.9% to 85.4±6.2% in the single-room world and from 77.0±6.4% to 84.0±9.2% in the three-room world, at a cost of one to two additional scans on average (single-room 2.0±0.0→3.6±0.9; multi-room 2.3±0.5→3.6±0.7); see Figure 8 and Table 2. To bound what is attainable along these paths, we also replay a uniform no-skip policy that scans at every candidate: it reaches 91.9±7.0% LOS (single-room) and 86.6±9.9% (multi-room), but at 28.0 and 40.8 scans on average. The visibility rule thus comes within 6.5 and 2.6 percentage points of this brute-force upper reference while taking roughly an eighth as many scans, and exceeds the disk baseline by +8.4 and +7.0 percentage points for one to two additional scans. On paths that traverse every room, where the disk rule’s blindness to occlusion is fully exercised, the per-path coverage gain reaches +21 to +23 percentage points. The disk spacing rule is structurally capped: once two scans are placed, almost every later candidate lies within *R* (straight line) of a prior scan, so it stops scanning even though walls occlude much of the area it counts as covered.

The disk marginal-gain baseline isolates the coverage model from the acceptance rule. Under the identical dual criterion of Section 4, the disk model accepts 2.6±0.5 scans for 77.7±3.8% LOS in the single-room world—essentially the spacing rule’s result—and 2.9±0.3 scans for only 59.1±1.3% LOS in the multi-room world, markedly *worse* than even the spacing rule. The mechanism is instructive. Because $B_{\mathrm{disk}}$ claims area through partitions, the disk model’s own coverage bookkeeping saturates after the first few acceptances: every later candidate appears almost fully covered, so the rule stops scanning while whole rooms remain unobserved. The spacing rule, blind to coverage altogether, at least keeps accepting whenever the robot moves *R* away from all prior scans and thus occasionally scans another room by accident. That the same acceptance rule produces a 24.9-point LOS gap between the two coverage models in the multi-room world (59.1% versus 84.0%) is the sharpest form of this paper’s claim: the deficiency lies in the disk coverage model itself, not in the acceptance rule attached to it.

### 6.3. Live Multi-Room Demonstration (Qualitative)

**Figure 9:**
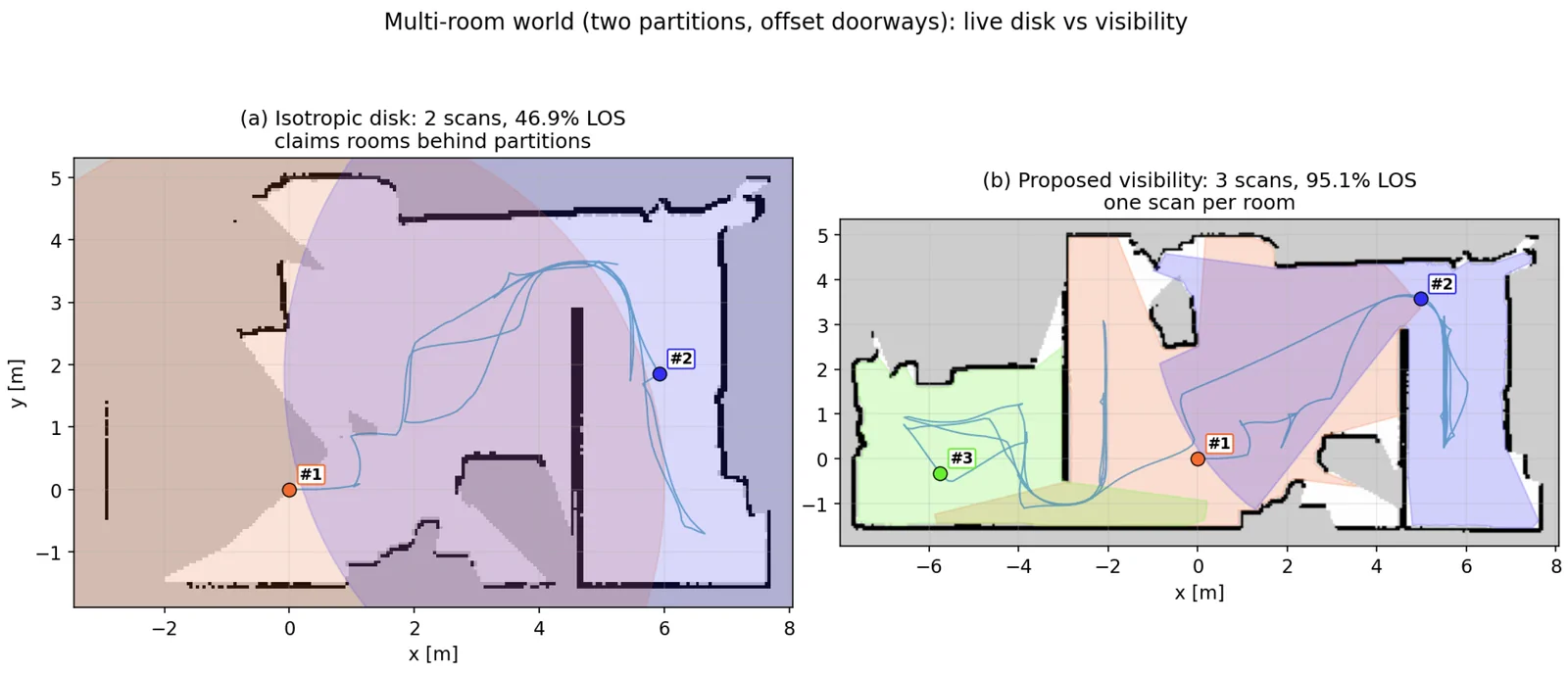
Multi-room world (two partitions, offset doorways): live disk vs. visibility. (**a**) The disk model skips candidates within *R* of a prior scan through the partitions, giving 2 scans and 46.9% LOS with a whole room unscanned. (**b**) The visibility model scans once per room, giving 3 scans and 95.1% LOS. Markers #1–#3 and colors index the scan order, as in Figure 5.

Figure 9 shows a single live autonomous run on the multi-room world under each coverage model. The disk policy stops after two scans at 46.9% LOS, having counted an entire room behind a partition as covered through the wall; the visibility policy places one scan per room, three in total, and reaches 95.1% LOS. We present this run as a qualitative demonstration rather than a statistical result: in live runs on the partitioned world, which rooms get mapped (a function of frontier selection and navigation through narrow, offset doorways) varies from run to run and dominates the measured coverage largely independently of the skip rule, which inflates variance and shrinks the apparent gap. The rigorous, confound-free claim is, therefore, the controlled ablation above; the live run illustrates how that difference manifests in a complete mission.

### 6.4. Coverage Completion

**Figure 10:**
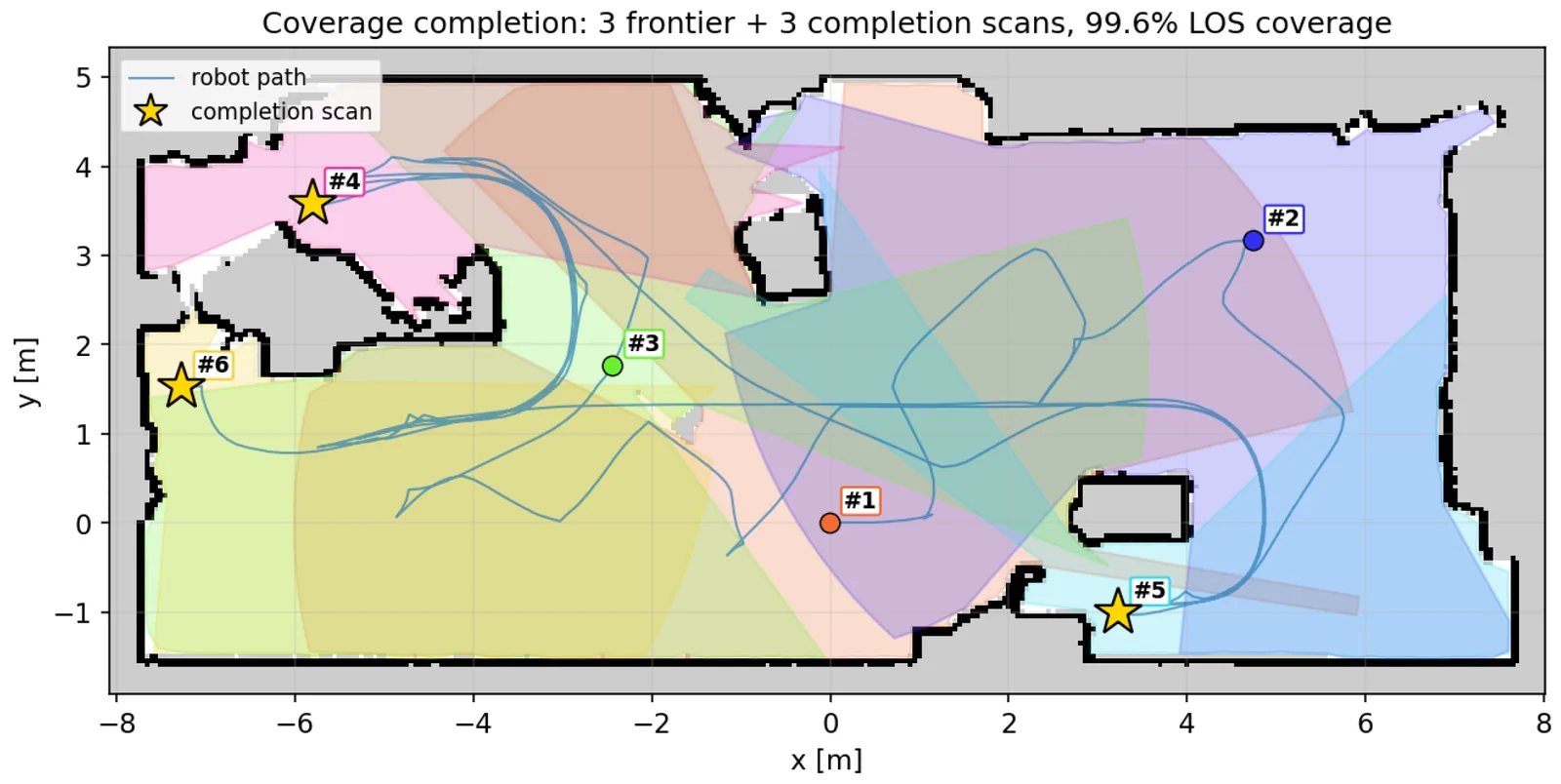
Coverage completion. After frontier exploration, the robot drives into each still-uncovered pocket and scans (★) until none above the minimum pocket area remains. Here 3 frontier + 3 completion scans, 99.6% LOS. Markers #1–#6 and colors index the scan order, as in Figure 5.

With the coverage-completion phase enabled, the robot continues after frontier convergence to drive into and scan the remaining uncovered pockets. Over N=5 runs this raises LOS coverage to 98.9±0.9% using 5.0±1.2 total scans on average (3.2 frontier plus 1.8 completion scans), with no unreachable pockets across the five runs. A representative run reaches 99.6% LOS with three frontier scans followed by three completion scans (Figure 10). The method does not claim a full 100%: residual fragments smaller than the minimum pocket area at wall and obstacle edges are reported as remaining.

### 6.5. Parameter Sensitivity

**Figure 11:**
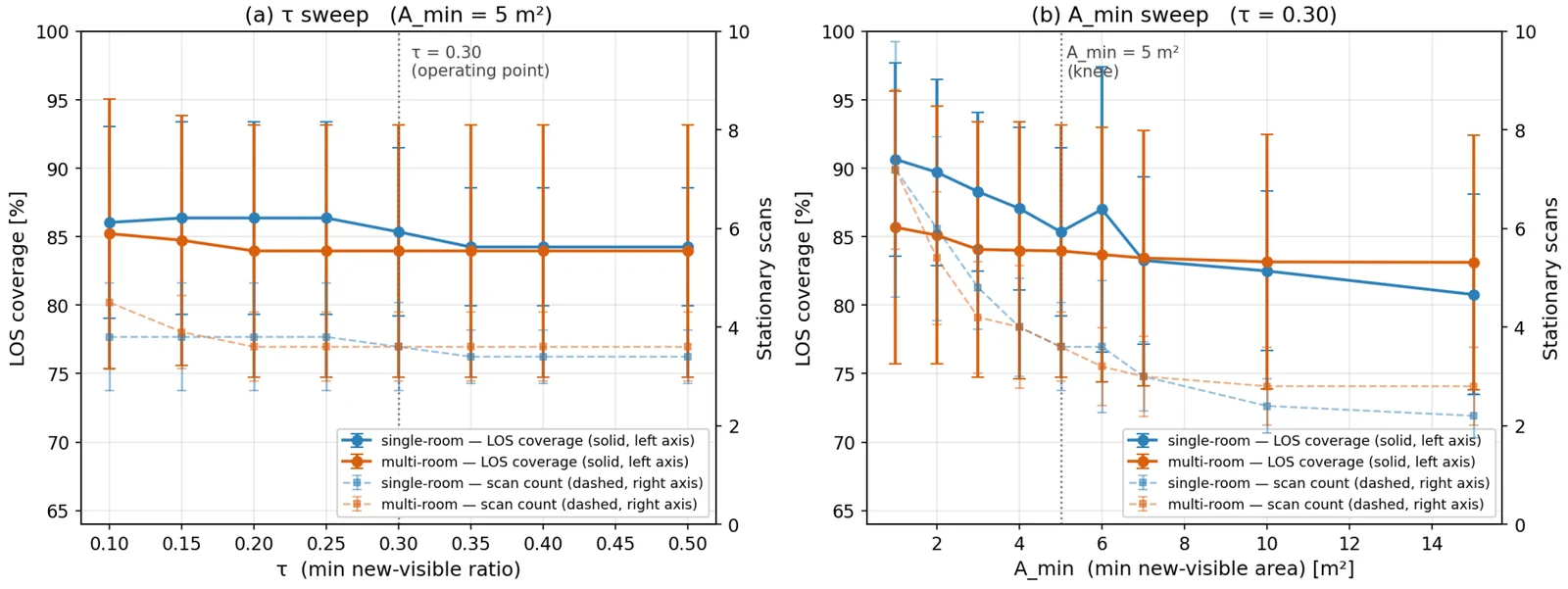
Parameter sweep by deterministic replay on the fixed candidate paths (mean ± s.d. over N=5 single-room and N=10 multi-room paths). Solid curves: LOS coverage (left axis); dashed curves: scan count (right axis). (**a**) τ sweep at $A_{\min}=5$ m^2^; (**b**) $A_{\min}$ sweep at τ=0.30, knee marked.

We characterize the two skip thresholds by deterministic replay of the visibility policy over the same fixed paths as the ablation (N=5 single-room, N=10 multi-room paths); Figure 11 reports means with per-path standard deviations as error bars. Varying the relative threshold τ over [0.10,0.50] changes mean LOS coverage by at most about two points (single-room 86.4→84.3%; multi-room 85.2→84.0%) and mean scan count by less than one scan, so τ lies in a robust, flat region and τ=0.30 is not a sensitive choice. The absolute threshold $A_{\min}$ is the operative knob that trades coverage against scan count. We identify $A_{\min}=5$ m^2^ as the knee by the following criterion: it is the largest threshold whose mean LOS remains within about five points of the most aggressive setting ($A_{\min}=1$ m^2^) in both environments (single-room 85.4 versus 90.7%; multi-room 84.0 versus 85.7%) while already halving the scan count (3.6 versus 7.2 scans in both environments). Beyond the knee the trade turns unfavorable: pushing to $A_{\min}=15$ m^2^ saves only about one further scan (3.6→2.2 and 3.6→2.8) while coverage continues to erode (85.4→80.8% and 84.0→83.1%). This justifies the operating point τ=0.30, $A_{\min}=5$ m^2^, which was fixed before the hardware campaign.

### 6.6. Fully Autonomous On-Hardware Disk-Versus-Visibility Comparison

**Figure 12:**
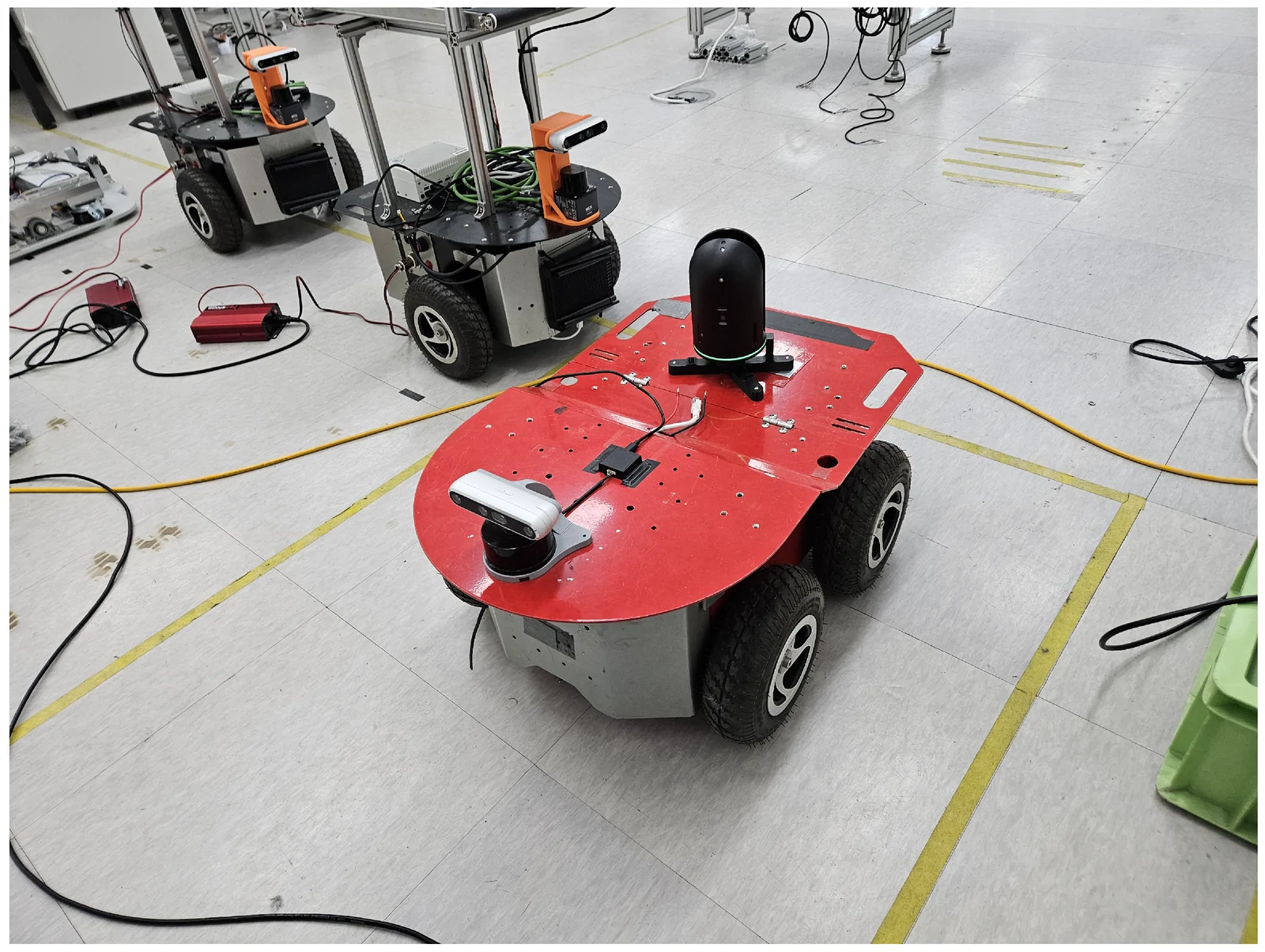
RedBOT04 deployed in the industrial test room during the fully autonomous on-hardware runs, with the Leica BLK360 G1 mounted on its deck. The platform navigates autonomously between scan stations and halts at each accepted pose to acquire a stationary full-dome scan.

The preceding results characterize the placement policy in simulation. To test whether this behavior carries over to the physical platform, we conducted a fully autonomous on-hardware comparison in the industrial test room of Figure 4: RedBOT04 explores the room live under frontier exploration through the full Nav2 stack (MPPI controller) while Cartographer builds the map online from the onboard 2D LiDAR, and the stop-and-scan sequencer applies the placement policy of Section 4 in real time. At each accepted pose, the platform halts and the deck-mounted Leica BLK360 G1 acquires a full-dome stationary scan (Figure 12). Each run is driven end to end by one skip rule—disk or visibility—so the comparison exercises the entire pipeline, perception through placement through scan acquisition, on real hardware. This is the same software stack used throughout the simulation study, now running on the physical robot.

**Figure 13:**
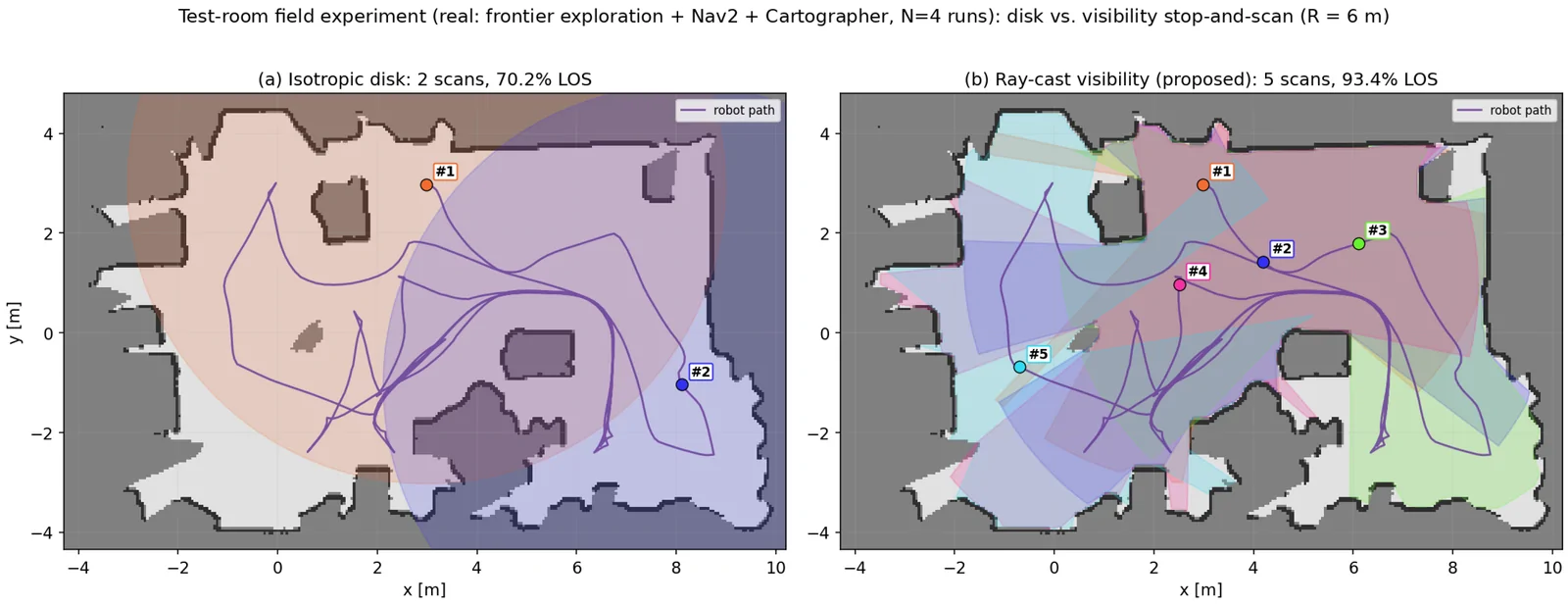
Fully autonomous on-hardware comparison in the physical test room (representative runs; R=6 m). Light grey: mapped free space; dark grey: unmapped; black: occupied; purple line: autonomous robot path; colored markers: placed scans with their coverage regions. (**a**) Disk spacing rule; (**b**) visibility rule. Markers #1–#5 and colors index the scan order, as in Figure 5.

Of the on-hardware exploration runs, we retain the N=4 per policy (eight completed runs in total) that completed a room-spanning traverse; two additional early-terminated runs, in which frontier exploration stalled shortly after start, are discarded as failed trials rather than policy samples. The exclusion criterion is independent of the skip rule: a run is discarded only when frontier exploration stalls before a room-spanning traverse begins, i.e., before the placement policy has faced any consequential scan/skip decision. Both discarded runs stalled in this stage and are, therefore, uninformative about either skip rule. Because each live run maps a slightly different extent of free space, we follow the common-reference protocol of Section 5: every run is evaluated on the single most complete SLAM map among the runs, so all runs share one free-space denominator. Unlike the simulated ablation, hardware runs cannot be paired—each is an independent live trial under one rule—so this experiment complements the confound-free paired ablation with ecological validity rather than replacing it. Table 3 reports the aggregate and Figure 13 a representative run.

The outcome reproduces the controlled ablation on the physical platform—a platform kinematically and dimensionally different from the simulated base, indicating that the effect is a property of the coverage model rather than of any particular robot. The isotropic disk over-skips: it places only 1.8±0.5 scans and, treating floor behind the equipment island and furniture as covered, attains 77.6±10.5% LOS coverage with a large run-to-run variance, since whether its few stations happen to see the occluded bays is largely incidental. The visibility rule recognizes the occluded area as unseen, places 4.2±1.5 scans, and covers 92.1±3.8% of the free space reliably—a far smaller run-to-run variance at the cost of about two extra scans. Whether the scans these autonomous runs produce actually register at survey grade is validated next (Section 6.7).

### 6.7. Survey-Grade Registration of the Autonomously Acquired Scans

The on-hardware comparison establishes where each rule scans; the remaining question is whether the scans each rule produces meet the survey-grade premise—that stationary scans placed to preserve line-of-sight overlap register at survey grade on real data. To answer it, we registered the BLK360 scans of every completed run offline, each run as an independent project under identical settings, in Leica Cyclone REGISTER 360 PLUS (BLK Edition; Leica Geosystems AG, Heerbrugg, Switzerland; https://leica-geosystems.com, accessed on 23 August 2026) using cloud-to-cloud alignment without artificial targets. Registration is a post-mission batch step; importing and aligning one run’s network took on the order of minutes on a desktop workstation, small compared with the acquisition time of the scans themselves. Table 4 summarizes the per-run registration networks.

**Figure 14:**
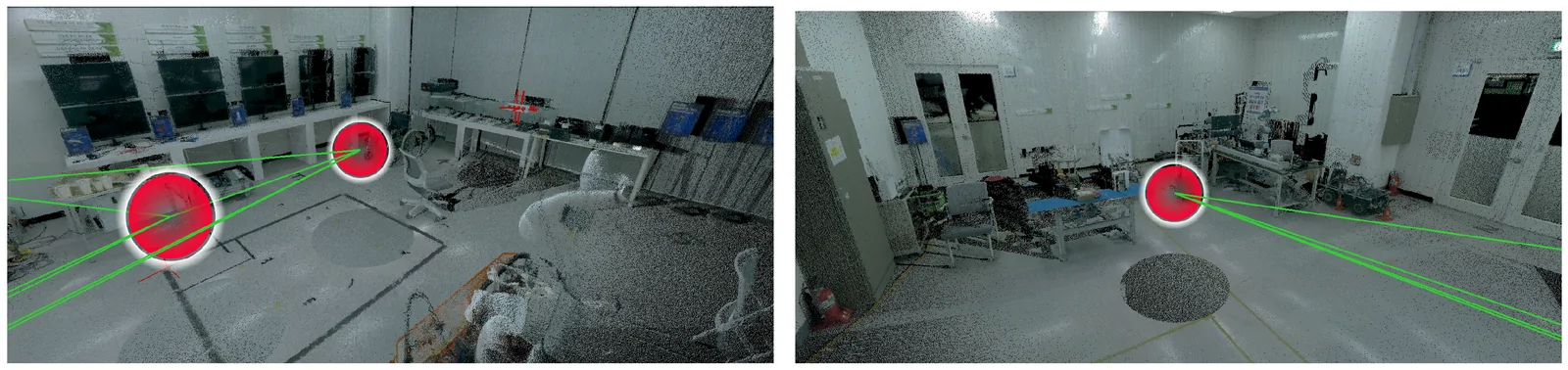
Interior views of the registered colorized point cloud of visibility run 3 along two diagonal directions: toward the display wall (**left**) and toward the equipment side (**right**). Red markers and green edges are the scan stations and links of Table 4.

**Figure 15:**
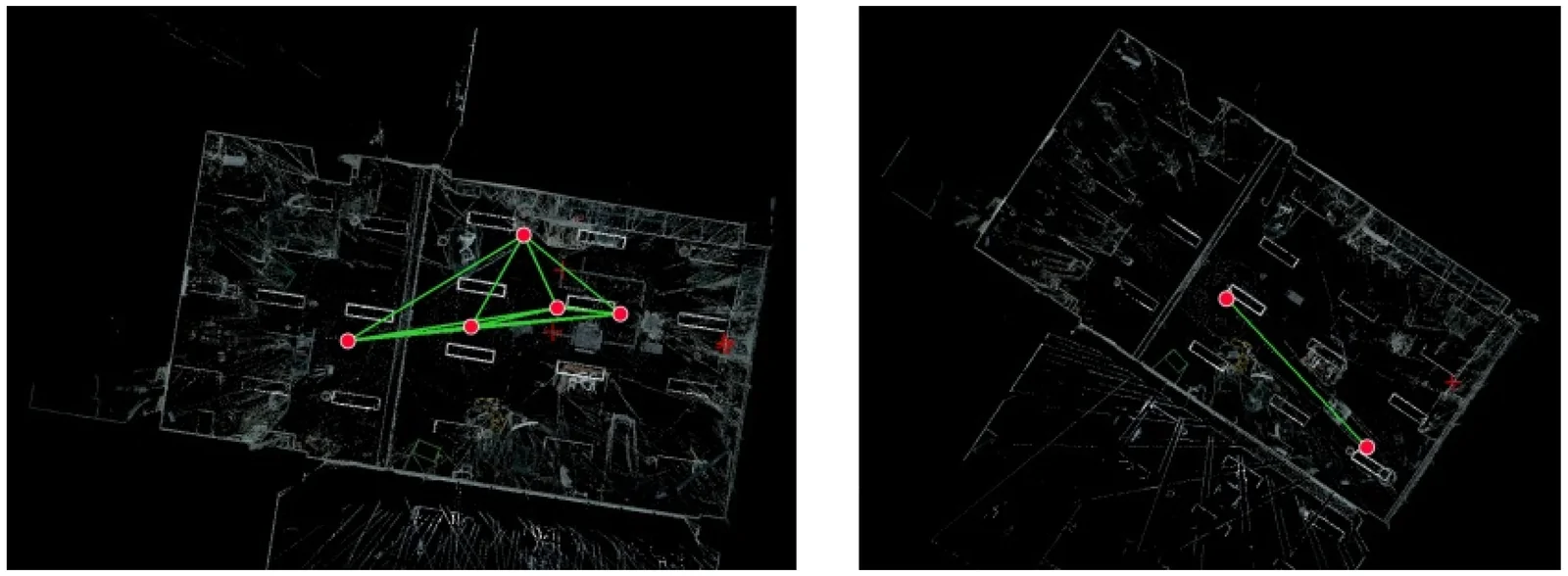
Registered scan-station networks (top-down view; red markers are setup positions, green edges are cloud-to-cloud links of Table 4). (**Left**) Visibility run 3: five setups joined by ten redundant links reconstruct the room as a single coherent model. (**Right**) Disk run 4: two setups joined by a single link—a minimal network with no redundant constraint, covering only part of the room.

All four visibility runs registered at survey grade. Their networks comprise 3–6 setups joined by 3–13 pairwise links, the bundle adjustments converged at 4–5 mm with overall overlaps of 84–88%, and every individual link fell within the software’s highest quality band (maximum error ≤15 mm; per-link overlaps 74–92%, errors 3–6 mm). The 4–5 mm bundle residuals are of the same order of magnitude as the scanner’s stated single-setup 3D point accuracy of a few millimeters. The registered clouds reconstruct the room—display wall, equipment, and floor—as a single coherent model: Figure 14 shows interior views of the registered cloud of visibility run 3 along two diagonal viewing directions, and Figure 15 (top) the corresponding scan-station network.

The disk runs tell a structurally different story. The three disk runs that placed two scans each registered as a two-setup network with a single link (75–85% overlap, 2 mm); the fourth disk run placed only one scan and, therefore, admits no registration at all. The low single-link residuals should not be read as superior registration: with one link there is no redundancy, so the alignment is not cross-checked by any independent constraint, and the resulting model covers correspondingly less of the room (Section 6.6). The structural contrast is the point: the visibility rule reliably produces redundant, bundle-verifiable multi-link networks, whereas the disk rule yields at best a minimal single-link pair and at worst a single unregistrable setup (Figure 15).

These results validate the workflow end to end on real hardware and confirm the overlap rationale that motivates the visibility model: placement that keeps successive stations in mutual line of sight consistently yields pairwise overlaps well above the level at which cloud-to-cloud registration is reliable, and the resulting bundles are survey grade in every visibility run. One remark bounds the scope: bundle strengths are moderate (38–48%) across runs, reflecting the corridor-like traverses the explorer takes through the room; a loop-closing or areal station layout would raise this redundancy measure, and the placement policy’s coverage-completion phase is a natural driver of such layouts. Together with Section 6.6, this completes the chain of evidence on the physical room: the live comparison establishes that the visibility rule, not the disk rule, reliably produces high-LOS scan placements, and the per-run registration establishes that those placements consistently yield survey-grade, bundle-verifiable scan networks—while disk placements do not even guarantee a registrable network. A downstream validation in which an independent robot navigates the completed map to quantify localization accuracy remains as future work.

## 7. Discussion

The controlled ablation isolates a single design choice, the coverage model used for the scan/skip decision, and shows that occlusion-aware visibility yields consistently higher LOS coverage than the Euclidean disk at the cost of one to two additional scans per environment; the disk baseline run under the identical marginal-gain rule performs even worse than the spacing rule in the multi-room world, attributing the deficiency to the coverage model itself rather than to the acceptance rule. The effect is structural rather than incidental: the disk model is provably optimistic ($B\subseteq B_{\mathrm{disk}}$ always holds, with equality only in obstacle-free surroundings), so wherever a wall lies within range it over-claims coverage and suppresses a needed scan. The live multi-room run makes the practical consequence vivid: counting a room through a partition caps the disk policy at 46.9% LOS, while the visibility policy scans each room. The same pattern reappears on the physical platform: in the fully autonomous on-hardware comparison in the industrial test room (Section 6.6), the disk rule attains 77.6±10.5% LOS with large run-to-run variance—whether its few stations happen to see the occluded bays is incidental—whereas the visibility rule reaches 92.1±3.8% reliably, indicating that the effect is not an artifact of simulation. Per-run registration of the acquired scans (Section 6.7) extends the contrast to the survey product itself: every visibility run yields a redundant, bundle-verifiable network at 4–5 mm, while the disk rule’s sparse placements yield at best a single uncorroborated link and at worst no registrable network at all.

Several limitations frame the result. First, the planning representation is two-dimensional: visibility is evaluated on the occupancy grid at sensor height, so vertical occlusions—shelves, overhangs, elevated machinery, or surfaces stacked at different heights above the same floor cell—are not modeled, and LOS coverage certifies floor-space sight lines rather than full 3D surface coverage (Section 3.5). The robotic deployment adds a related height effect: the deck-mounted scanner observes from roughly 0.5 m above the floor, lower than a typical tripod setup, so low furniture and equipment cast longer horizontal shadows than in a manual survey of the same room. This makes occlusion-aware placement more important on a robotic platform, not less; but it also means the acquired clouds have a lower vantage than a conventional tripod survey would provide. Extending the model to a genuinely 3D visibility representation, for example, on a voxel or octree map or over reconstructed surfaces, is the principal direction for future work. Second, even within its 2D scope, LOS coverage is a geometric proxy for observation quality: it certifies an unobstructed sight line within the range bound but does not model incidence angle, range-dependent point density, or surface reflectivity, all of which affect the usefulness of a survey scan. A visibility-weighted coverage that discounts grazing or far-range cells is a natural refinement. Third, the controlled paired ablation is conducted in simulation with a mock scanner, because hardware runs cannot be paired: each live run sees its own map and trajectory. The real-hardware experiments narrow this gap from two directions—the fully autonomous comparison of Section 6.6 reproduces the placement gain with each rule driving the physical robot end to end, and Section 6.7 registers every run’s scans independently, showing survey-grade networks in all visibility runs—but the on-hardware comparison is by nature live and unpaired, so these results complement rather than replace the confound-free ablation. A downstream navigation validation, in which an independent robot localizes against the completed map to quantify end-use accuracy, is left to future work. Fourth, the visibility model presently assumes an optical line-of-sight sensor, which is appropriate for a TLS but not for the penetrating or dwell-time sensors that share the stop-and-scan workflow.

This last point also indicates a path to generalization. The coverage model treats the sensor as a fixed optical ray-caster, but the stop-and-scan family spans sensors with very different observation models. A promising direction is to attach a sensor-level semantic description, for example whether a sensor penetrates obstacles and over what range and field of view, to the robot model, and to let that description parameterize the ray-termination rule so that the same placement policy adapts across heterogeneous stop-and-scan sensors without code changes. Populating such descriptions automatically, e.g., from sensor datasheets, and the downstream navigation validation on the completed map are the focus of ongoing work.

## 8. Conclusions

We presented an occlusion-aware visibility coverage model for robotic stop-and-scan 3D LiDAR mapping, together with a marginal-gain placement policy built on it; the model performs 2D scan-station planning on the live occupancy map for subsequent 3D mapping, and a survey-grade terrestrial laser scanner is its experimental instantiation. Replacing the isotropic disk with a ray-cast visibility region and scoring candidates by line-of-sight marginal gain corrects the disk model’s structural over-claiming of occluded space. In a physics-based simulation built from a real scanner-derived environment, a controlled paired ablation that isolates the skip decision showed that the visibility rule raises LOS coverage from about 77% to 84–85% at the cost of one to two additional scans, while a disk baseline governed by the identical marginal-gain rule performed no better than the spacing rule in the single-room world and markedly worse in the multi-room world (59.1% LOS), attributing the deficiency to the coverage model itself; a live multi-room run improved coverage from 46.9% to 95.1% relative to the disk model, and an optional coverage-completion phase reached about 99% LOS. A parameter study showed the relative threshold is robust while the absolute-area threshold is the operative coverage–scan trade-off knob. Beyond simulation, a fully autonomous on-hardware comparison in an industrial test room, with each skip rule driving the live exploration and scan acquisition of a four-wheeled platform carrying a BLK360 G1, reproduced the placement gain (77.6→92.1% LOS) on the physical robot; per-run offline registration showed that every visibility run yields a survey-grade, redundant multi-link network (4–5 mm bundle error, 84–88% overlap), whereas disk runs yield at best a single-link network and at worst a single unregistrable scan. Future work will refine LOS into a visibility-weighted coverage that accounts for incidence and range, generalize the ray-termination rule to heterogeneous stop-and-scan sensors via sensor-level semantic descriptions, extend the coverage model to a genuinely 3D visibility representation (voxel-, octree-, or surface-based) that captures vertical occlusion, and add a downstream navigation validation in which an independent robot autonomously localizes against the completed map. Within the room-scale indoor environments and the optical stop-and-scan sensor class studied here, the evidence supports occlusion-aware visibility, rather than Euclidean proximity, as the basis for deciding where a robot should stop and scan.

## Figures and Tables

**Table 1:** Principal parameters of the stop-and-scan mapping subsystem. Values in the upper block are the operating point used in all experiments unless otherwise noted; values in the lower block are tuned per environment.

Parameter	Value	Description
Range bound *R*	6 m	Maximum distance out to which the visibility region B(s,R) is evaluated.
Relative threshold τ	0.30	Minimum fraction of new visible area for the relative accept criterion.
Absolute threshold $A_{\min}$	5 m^2^	Minimum new visible area for the absolute accept criterion.
Candidate interval *d*	3 m	Net straight-line displacement between successive scan candidates.
Ray count *K*	720	Number of rays per visibility computation (Δθ=0.5°).
Free threshold $\theta_{\mathrm{free}}$	25	Upper occupancy value for a cell to count as mapped free space.
Occupied threshold $\theta_{\mathrm{occ}}$	65	Lower occupancy value for a cell to terminate a ray as an obstacle.
Degenerate cut	0.5 m^2^	Minimum visibility-region area; smaller candidates are rejected.
Scan-and-download time	env.-tuned	Mock-scanner capture-plus-download duration matching the BLK360 G1 cycle.
Bilateral-filter std. dev.	env.-tuned	Spatial and range Gaussian kernel widths for decision-map construction.
Free-space dilation radius	env.-tuned	Morphological dilation of mapped free space during decision-map construction, before frontier extraction.
Minimum frontier size	env.-tuned	Frontier clusters smaller than this are discarded as noise.
Completion pocket area $A_{\mathrm{pocket}}$	env.-tuned	Minimum uncovered-pocket area triggering a completion scan.

**Table 2:** Controlled skip-rule ablation: four placement policies replayed on the same fixed candidate sequences (N=5 single-room paths; N=10 multi-room paths). Values are mean ± s.d. The uniform no-skip policy is an upper reference on the LOS attainable along the path, at a prohibitive scan count; the disk marginal-gain row applies the identical accept/skip rule as the visibility policy, so within each environment that pair differs only in the coverage model.

Environment	Coverage Model	LOS Coverage (%)	Scans
Single-room	Uniform, no skip (reference)	91.9±7.0	28.0±13.0
	Disk, spacing rule (baseline)	77.0±4.9	2.0±0.0
	Disk, marginal-gain rule	77.7±3.8	2.6±0.5
	Ray-cast visibility (ours)	85.4±6.2	3.6±0.9
Multi-room	Uniform, no skip (reference)	86.6±9.9	40.8±11.8
	Disk, spacing rule (baseline)	77.0±6.4	2.3±0.5
	Disk, marginal-gain rule	59.1±1.3	2.9±0.3
	Ray-cast visibility (ours)	84.0±9.2	3.6±0.7

On paths that traverse every room, the per-path gain of the visibility rule over the disk spacing rule reaches +21 to +23 percentage points; the means above are over all paths, including partial-traversal paths. All policies traverse the identical candidate path, so total travel distance is the same across rows; the policies differ only in scan count, the dominant cost in stop-and-scan sensing. Bold indicates the best LOS coverage in each environment.

**Table 3:** Fully autonomous on-hardware disk-versus-visibility comparison in the physical test room (N=4 completed live frontier-exploration runs per policy on RedBOT04, each driven end to end by one skip rule; all runs evaluated on the common reference map). Values are mean ± s.d.

Coverage Model	Scans	LOS Coverage (%)
Isotropic disk (baseline)	1.8±0.5	77.6±10.5
Ray-cast visibility (ours)	4.2±1.5	92.1±3.8

BLK360 scans were acquired at every accepted pose in all runs; the per-run offline registration of these scans is reported in Section 6.7. Bold indicates the best LOS coverage.

**Table 4:** Per-run offline registration of the autonomously acquired scans (Leica Cyclone REGISTER 360 PLUS, BLK Edition; cloud-to-cloud, no targets, identical settings). Every link of every registered network lies within the software’s highest quality band (maximum error ≤15 mm).

Policy	Run	Setups	Links	Overall Overlap (%)	Bundle Error (mm)
Visibility	1	3	3	86	5
Visibility	2	6	13	86	5
Visibility	3	5	10	84	5
Visibility	4	3	3	88	4
Disk	1	1	0	not registrable (single setup)	
Disk	2	2	1	75	2
Disk	3	2	1	85	2
Disk	4	2	1	84	2

## Data Availability

The source code and simulation assets supporting this study are available from the corresponding author upon reasonable request and will be released publicly upon publication.

## Abbreviations

The following abbreviations are used in this manuscript:

TLS	Terrestrial laser scanner
LiDAR	Light detection and ranging
LOS	Line of sight
NBV	Next-best-view
SLAM	Simultaneous localization and mapping
MPPI	Model predictive path integral
GPR	Ground-penetrating radar
BIM	Building information model

## References

[1] Leica Geosystems (2022). Leica BLK360 Imaging Laser Scanner: Product Specifications.

[2] Lin Y., Hyyppä J., Kukko A. (2013). Stop-and-Go Mode: Sensor Manipulation as Essential as Sensor Development in Terrestrial Laser Scanning. Sensors.

[3] Arain M.A., Trincavelli M., Cirillo M., Schaffernicht E., Lilienthal A.J. (2015). Global Coverage Measurement Planning Strategies for Mobile Robots Equipped with a Remote Gas Sensor. Sensors.

[4] Halder S., Afsari K. (2023). Robots in Inspection and Monitoring of Buildings and Infrastructure: A Systematic Review. Appl. Sci..

[5] Placed J.A., Strader J., Carrillo H., Atanasov N., Indelman V., Carlone L., Castellanos J.A. (2023). A Survey on Active Simultaneous Localization and Mapping: State of the Art and New Frontiers. IEEE Trans. Robot..

[6] Yamauchi B. A frontier-based approach for autonomous exploration. Proceedings of the IEEE International Symposium on Computational Intelligence in Robotics and Automation (CIRA).

[7] Umari H., Mukhopadhyay S. Autonomous robotic exploration based on multiple rapidly-exploring randomized trees. Proceedings of the IEEE/RSJ International Conference on Intelligent Robots and Systems (IROS).

[8] Galceran E., Carreras M. (2013). A survey on coverage path planning for robotics. Robot. Auton. Syst..

[9] Choset H. (2001). Coverage for robotics—A survey of recent results. Ann. Math. Artif. Intell..

[10] Kim S., Kuc T.-Y. Coverage-Aware Stop-and-Scan Frontier Exploration for Automated Survey-Grade 3D Mapping. Proceedings of the 2026 International Conference on Control, Automation and Systems (ICCAS).

[11] Lluvia I., Lazkano E., Ansuategi A. (2021). Active Mapping and Robot Exploration: A Survey. Sensors.

[12] Connolly C. The determination of next best views. Proceedings of the IEEE International Conference on Robotics and Automation (ICRA).

[13] Bircher A., Kamel M., Alexis K., Oleynikova H., Siegwart R. Receding horizon “next-best-view” planner for 3D exploration. Proceedings of the IEEE International Conference on Robotics and Automation (ICRA).

[14] Stachniss C., Grisetti G., Burgard W. Information gain-based exploration using Rao-Blackwellized particle filters. In Proceedings of Robotics: Science and Systems (RSS).

[15] Burgard W., Moors M., Stachniss C., Schneider F.E. (2005). Coordinated multi-robot exploration. IEEE Trans. Robot..

[16] Liu C., Zhang D., Liu W., Sui X., Huang Y., Ma X., Yang X., Wang X. (2025). Enhancing autonomous exploration for robotics via real-time map optimization and improved frontier costs. Sci. Rep..

[17] Quin P., Nguyen D.D.K., Vu T.L., Alempijevic A., Paul G. (2021). Approaches for Efficiently Detecting Frontier Cells in Robotics Exploration. Front. Robot. AI.

[18] Zhou B., Zhang Y., Chen X., Shen S. (2021). FUEL: Fast UAV Exploration Using Incremental Frontier Structure and Hierarchical Planning. IEEE Robot. Autom. Lett..

[19] Schmid L., Pantic M., Khanna R., Ott L., Siegwart R., Nieto J. (2020). An Efficient Sampling-Based Method for Online Informative Path Planning in Unknown Environments. IEEE Robot. Autom. Lett..

[20] González-Baños H.H., Latombe J.-C. (2002). Navigation Strategies for Exploring Indoor Environments. Int. J. Robot. Res..

[21] O’Rourke J. (1987). Art Gallery Theorems and Algorithms.

[22] Urrutia J., Sack J.-R., Urrutia J. (2000). Art gallery and illumination problems. Handbook of Computational Geometry.

[23] Scott W.R., Roth G., Rivest J.-F. (2003). View planning for automated three-dimensional object reconstruction and inspection. ACM Comput. Surv..

[24] Tarabanis K.A., Allen P.K., Tsai R.Y. (1995). A survey of sensor planning in computer vision. IEEE Trans. Robot. Autom..

[25] Zeng R., Wen Y., Zhao W., Liu Y.-J. (2020). View Planning in Robot Active Vision: A Survey of Systems, Algorithms, and Applications. Comput. Vis. Media.

[26] Almadhoun R., Taha T., Seneviratne L., Zweiri Y. (2019). A survey on multi-robot coverage path planning for model reconstruction and mapping. SN Appl. Sci..

[27] Park S., Ju S., Nguyen M.H., Yoon S., Heo J. (2024). Automated Point Cloud Registration Approach Optimized for a Stop-and-Go Scanning System. Sensors.

[28] Aryan A., Bosché F., Tang P. (2021). Planning for terrestrial laser scanning in construction: A review. Autom. Constr..

[29] Heidari Mozaffar M., Varshosaz M. (2016). Optimal placement of a terrestrial laser scanner with an emphasis on reducing occlusions. Photogramm. Rec..

[30] Prieto S.A., Quintana B., Adán A., Vázquez A.S. (2017). As-is building-structure reconstruction from a probabilistic next best scan approach. Robot. Auton. Syst..

[31] Dehbi Y., Leonhardt J., Oehrlein J., Haunert J.-H. (2021). Optimal Scan Planning with Enforced Network Connectivity for the Acquisition of Three-Dimensional Indoor Models. ISPRS J. Photogramm. Remote Sens..

[32] Knechtel J., Dehbi Y., Klingbeil L., Haunert J.-H. (2025). Simultaneous Planning of Standpoints and Routing for Laser Scanning of Buildings with Network Redundancy. ISPRS J. Photogramm. Remote Sens..

[33] Elfes A. (1989). Using occupancy grids for mobile robot perception and navigation. Computer.

[34] Thrun S., Burgard W., Fox D. (2005). Probabilistic Robotics.

[35] Hess W., Kohler D., Rapp H., Andor D. Real-time loop closure in 2D LIDAR SLAM. Proceedings of the IEEE International Conference on Robotics and Automation (ICRA).

[36] Güler M. (2026). frontier_exploration_ros2: A Modern C++ Frontier Exploration Package for ROS 2. Open-Source Software. https://github.com/mertgulerx/frontier_exploration_ros2.

[37] Macenski S., Martín F., White R., Ginés Clavero J. The Marathon 2: A Navigation System. Proceedings of the IEEE/RSJ International Conference on Intelligent Robots and Systems (IROS).

[38] Williams G., Drews P., Goldfain B., Rehg J.M., Theodorou E.A. Aggressive driving with model predictive path integral control. Proceedings of the IEEE International Conference on Robotics and Automation (ICRA).

[39] Koenig N., Howard A. Design and use paradigms for Gazebo, an open-source multi-robot simulator. Proceedings of the IEEE/RSJ International Conference on Intelligent Robots and Systems (IROS).

